# Low-rate smartphone videoscopy for microsecond luminescence lifetime imaging with machine learning

**DOI:** 10.1093/pnasnexus/pgad313

**Published:** 2023-09-27

**Authors:** Yan Wang, Sina Sadeghi, Alireza Velayati, Rajesh Paul, Zach Hetzler, Evgeny Danilov, Frances S Ligler, Qingshan Wei

**Affiliations:** Department of Chemical and Biomolecular Engineering, North Carolina State University, Raleigh, NC 27695, USA; Department of Chemical and Biomolecular Engineering, North Carolina State University, Raleigh, NC 27695, USA; Department of Chemical and Biomolecular Engineering, North Carolina State University, Raleigh, NC 27695, USA; Department of Chemical and Biomolecular Engineering, North Carolina State University, Raleigh, NC 27695, USA; Department of Chemical and Biomolecular Engineering, North Carolina State University, Raleigh, NC 27695, USA; Department of Chemistry, North Carolina State University, Raleigh, NC 27695, USA; Department of Biomedical Engineering, Texas A&M University, College Station, TX 77843, USA; Department of Chemical and Biomolecular Engineering, North Carolina State University, Raleigh, NC 27695, USA

**Keywords:** smartphone videoscopy, V-chopper, time-resolved, lifetime imaging, machine learning

## Abstract

Time-resolved techniques have been widely used in time-gated and luminescence lifetime imaging. However, traditional time-resolved systems require expensive lab equipment such as high-speed excitation sources and detectors or complicated mechanical choppers to achieve high repetition rates. Here, we present a cost-effective and miniaturized smartphone lifetime imaging system integrated with a pulsed ultraviolet (UV) light-emitting diode (LED) for 2D luminescence lifetime imaging using a videoscopy-based virtual chopper (V-chopper) mechanism combined with machine learning. The V-chopper method generates a series of time-delayed images between excitation pulses and smartphone gating so that the luminescence lifetime can be measured at each pixel using a relatively low acquisition frame rate (e.g. 30 frames per second [fps]) without the need for excitation synchronization. Europium (Eu) complex dyes with different luminescent lifetimes ranging from microseconds to seconds were used to demonstrate and evaluate the principle of V-chopper on a 3D-printed smartphone microscopy platform. A convolutional neural network (CNN) model was developed to automatically distinguish the gated images in different decay cycles with an accuracy of >99.5%. The current smartphone V-chopper system can detect lifetime down to ∼75 µs utilizing the default phase shift between the smartphone video rate and excitation pulses and in principle can detect much shorter lifetimes by accurately programming the time delay. This V-chopper methodology has eliminated the need for the expensive and complicated instruments used in traditional time-resolved detection and can greatly expand the applications of time-resolved lifetime technologies.

**Significance Statement**

The videoscopy-based virtual chopper (V-chopper) method in this work decouples the traditional time-resolved detection from expensive and complicated instruments such as mechanical optical choppers. This simple method allows to resolve a broad range of lifetime from microseconds to seconds using a consumer electronic device such as a smartphone at a low frame rate (30 frames per second [fps]). The developed miniaturized smartphone V-chopper microscopy system exhibits huge potentiality in lifetime measurement for various field applications such as point-of-care biosensing. The methodology can also be a universal method, which can be applied on benchtop sensors to resolve nanosecond or even faster fluorescence events in the future without the need for high-speed detectors or choppers.

## Introduction

Time-resolved techniques, including time-gated autofluorescence-free imaging and fluorescence lifetime detection, have drawn significant attention in the past decades (1, 2). By taking advantage of a long-lived luminescence probe (3–6), the high background scattering and autofluorescence in biological samples can be effectively removed using time-resolved luminescence detection. Moreover, coded luminescence lifetimes can be exploited for temporally multiplexed detection assays, enabling multichannel detection while minimizing crosstalk between detection channels, which is a common limitation for the spectral multiplexing method (7, 8). Therefore, the time-resolved detection and luminescence lifetime imaging has found a wide variety of applications, such as high-contrast, in vivo imaging of cells and tissues (9–11), detection of rare diseased cells and pathogenic microorganisms (12–14), ultrasensitive bioassays (15–17), and physiological sensing (e.g. pH and temperature) (18–20). A range of analytical instruments such as spectrometers, microscopes, and flow cytometers have been adapted to enable time-resolved luminescence measurements (21–26). However, most current systems for time-resolved and lifetime detection require complicated mechanical choppers to achieve high repetition rates or expensive equipment such as high-speed excitation sources and detectors such as photomultiplier tubes (PMT), streak cameras, and intensified charge-coupled device (CCD) cameras to provide the temporal resolution (26–29). The bulkiness and complexity of the current time-resolved instruments, therefore, have posed significant challenges to the broad access to this technology outside of well-equipped laboratories.

Portable and cost-effective time-resolved devices are promising platforms for point-of-care (POC) monitoring for medical, agricultural, and environmental applications. In particular, modern smartphone equipped with advanced process unit and camera modules is an emerging platform for field-portable time-gated or time-resolved detection (30). For instance, time-gated imaging has been adopted on the smartphone by capturing persistent postexcitation luminescence (31, 32). On the other hand, lifetime resolving and imaging can be achieved by exponentially fitting the pixel intensities in consequent time-gated frames over time on the smartphone (33–38). However, the temporal resolution of smartphone camera is very limited due to its low frame rate (typically 30 frames per second [fps]). Even though the frame rate can reach 60 fps or higher on some smartphone models, it is still inadequate to detect lifetimes in the range of submillisecond (ms). As such, for demonstration purposes, many previous smartphone-based time-gated platforms (31–34, 38) selected persistent luminescent phosphors with ultralong lifetimes of hundreds of milliseconds to seconds (s). Additional mechanical apparatus such as chopper and motorized turntable were necessarily included into the system to break the limitation of temporal resolution of the smartphone for the measurement of shorter lifetimes in the microsecond (µs) range (35–37). Recently, B. Xiong and Q. Fang reported a portable lifetime imaging system that can detect lifetimes in the subhundred microsecond range using a smartphone camera with an electronic rolling shutter (ERS). The lifetime was extracted by measuring the phase shift of fringe profiles captured by the smartphone camera with an ERS at 30 fps. However, the lifetime image is sensitive to fringe distortion and cannot provide high pixel-by-pixel resolution due to the method of lifetime calculation (frequency domain-based) (39).

Here, a cost-effective and miniaturized smartphone-based lifetime imager was developed for luminescence lifetime quantification on the microsecond time scale using a virtual chopper (V-chopper) concept combined with machine learning. The smartphone V-chopper system was integrated with a pulsed UV LED, a UV reflection mirror, and a 615 nm band-pass filter in a 3D-printed enclosure. The V-chopper mechanism used the video rate (30 fps) of the smartphone to record repeated luminescence decay cycles and a convolutional neural network (CNN) model to extract correct gated images with >99.5% accuracy from different modulation cycles for lifetime image reconstruction. The effect of light pulse frequency, duty cycle, smartphone frame rate, and exposure time was systematically studied both experimentally and theoretically. Under the optimal setting, the smartphone V-chopper system can resolve luminescence lifetime from Europium (Eu) complex dyes as short as 75 µs. This portable smartphone V-chopper system decoupled the traditional time-resolved detection from expensive and complicated instruments such as mechanical choppers and high-speed detectors, making lifetime measurements practical in resource-limited settings.

## Results

### Design of the smartphone V-chopper lifetime imaging device

**Fig. 1.**
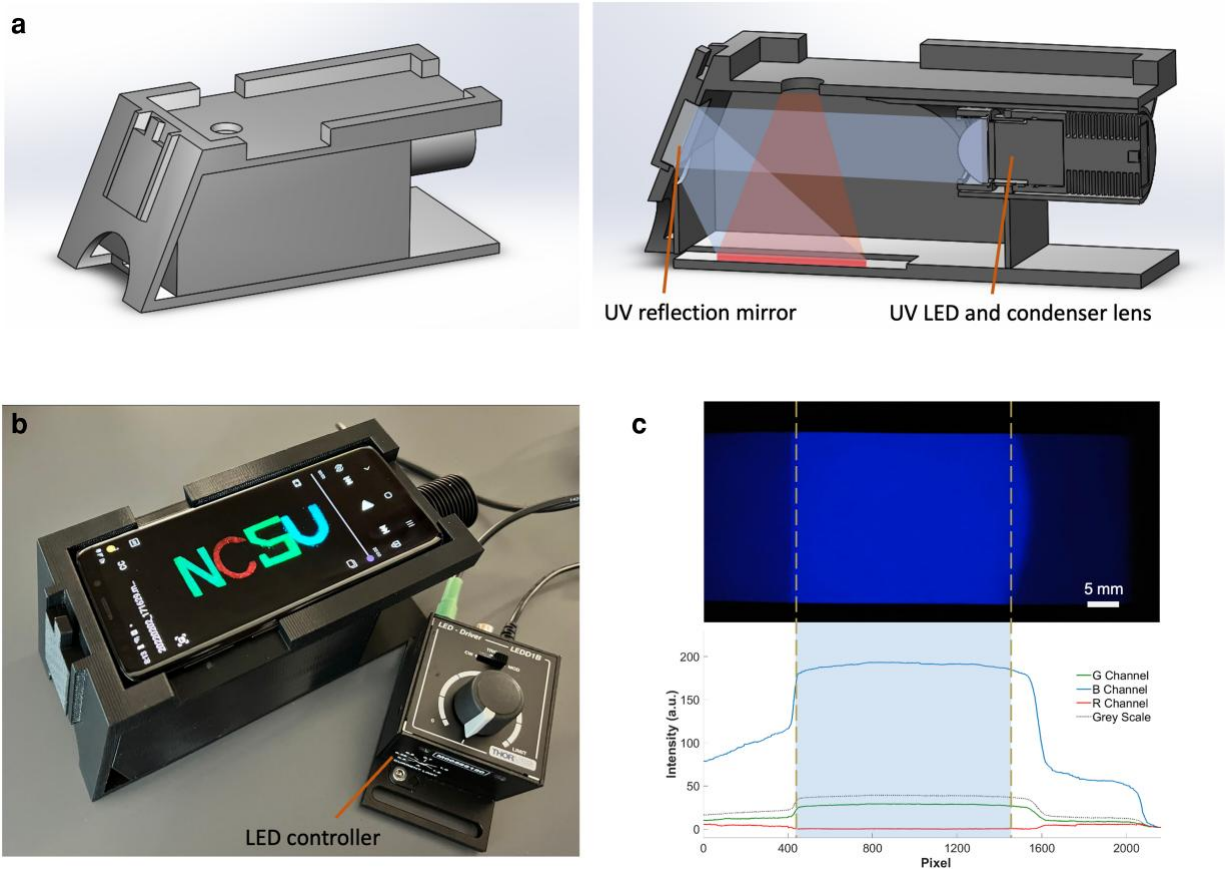
Design of the smartphone V-chopper lifetime system. a) Schematic and cross-section of the smartphone enclosure. b) Photograph of the actual smartphone V-chopper system with LED and pulse control. c) Illumination distribution of light from the LED on the sample slide.

The prototype for the smartphone V-chopper lifetime imaging device utilized a 3D-printed enclosure to integrate a UV LED (365 nm, M365L3, Thorlabs), a condenser lens (ACL2520U-A, Thorlabs), and a UV-enhanced reflection mirror (PFSQ10-03-F01, Thorlabs) with the smartphone camera (Fig. 1a and b). The UV LED was controlled by an LED driver (LEDD1B, Thorlabs) in the trigger or modulation mode and pulsed by a square wave voltage source (DG1062Z, Rigol). A 615 nm band-pass filter (87-739, Edmund Optics) was inserted in the enclosure in front of the phone camera when detecting luminescent signals from Eu complex dyes. The dyes were deposited on the glass slide, which was inserted at the bottom of the 3D-printed enclosure. A smartphone (Samsung Galaxy S9) with manual video control (e.g. ISO, focal length, shutter speed, video frame rate, and image resolutions) was placed on top of the enclosure as the detector. A 60 fps video frame rate was used for lifetime detection of ultralong luminescent materials (seconds). For the measurement of microsecond lifetime targets, a normal video rate (30 fps) was used combined with the V-chopper principle, as detailed below.

To deliver a uniform illumination to the sample slide, a 1” × 1” UV-enhanced mirror was used to reflect the excitation instead of direct illumination. The highly divergent emission from the UV LED was first collimated using the aspheric condenser lens (*f* = 20.1 mm, NA = 0.60) and then evenly projected on the glass slide by a tilted mirror (Fig. 1a). The tilted angle of the mirror is designed to be ∼67.5 degree relative to the slide surface, so that the illumination center is aligned with the field of view (35 × 63 mm^2^) of the smartphone. Figure 1c shows an autofluorescence image of a printing paper under UV excitation. The fluorescence intensity across the slide was extracted with Matlab for R, G, and B channels, respectively, showing the uniformity of the signals for subsequent measurements and experiments.

### Resolving ultralong lifetime in single decay cycle

**Fig. 2.**
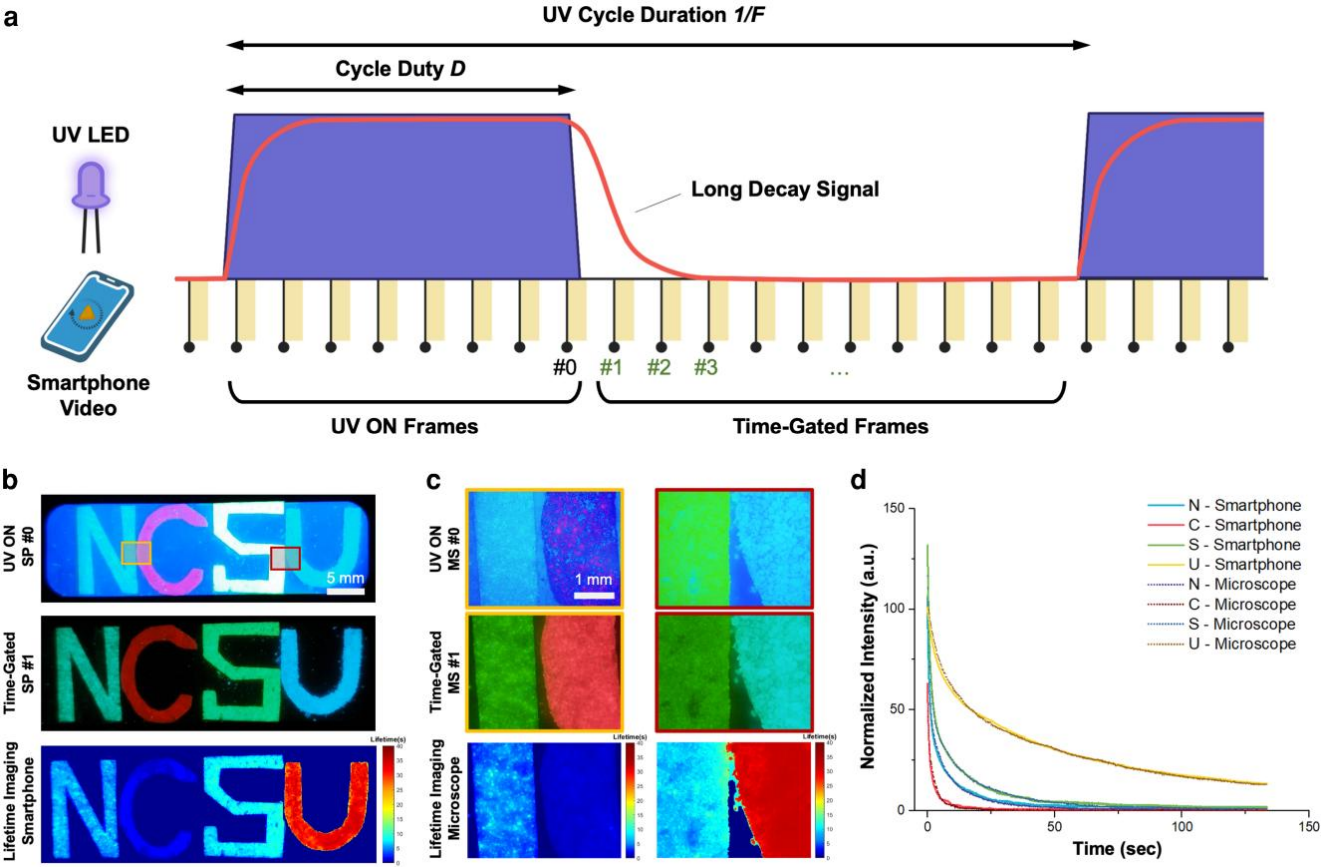
Smartphone-based lifetime imaging of ultralong (seconds) luminescent targets. a) Schematic of smartphone videoscopy for long lifetime measurement. Multiple gating can be applied to a single luminescence decay cycle. b) From Top to Bottom: smartphone fluorescence image (frame #0, UV on), smartphone time-gated image (frame #1, UV off), and smartphone lifetime image, respectively. c) From Top to Bottom: benchtop fluorescence images, benchtop time-gated images (frame #1, UV off), and benchtop lifetime images, respectively. Two different ROIs were selected from b (Top) for comparison. d) Luminescence decay curves extracted from the time-gated frames on smartphone (solid lines) and benchtop (dashed lines) microscopes, respectively.

We first tested the smartphone device for lifetime imaging of persistent luminescent probes by acquiring multiple gated images per decay cycle (Fig. 2a). The ultralong or persistent luminescent phosphors can glow for a relatively long time from seconds to even days after switching off excitation sources. The long luminescence is also called “afterglow” (40). Four afterglow composite powders of calcium sulfide (red) and strontium aluminate europium dysprosium (malachite, jade green, and cyan) were used as the testing samples and evenly dispensed on the adhesive tape, forming four letters “N,” “C,” “S,” and “U,” respectively (Fig. 2b). The glowing letters were sandwiched with glass slides and then inserted into the smartphone enclosure for lifetime imaging. The letters were excited for 10 s (0.005 Hz and 5% duty cycle) with an irradiance of 1 mW/cm^2^. No band-pass filter was used in this application in order to capture all four colors in the visible wavelength range. The smartphone took a video recording of both UV on (10 s) and UV off (∼190 s) time periods at a frame rate of 60 fps and exposure time of 1/60 s (Video S1). The image frames were then extracted from the recorded video by Matlab, and the time-gated frames were identified based on the background autofluorescence level. The last frame when the UV was on was assigned as the #0 frame (Fig. 2b, top), and the first frame after UV off was labeled as the #1 frame, which is also the first time-gated image in the gating cycle (Fig. 2b, middle). The delay time equals the integer times of the frame interval which is the reciprocal of frame rate (e.g. 1/60 s for frame #1, 2/60 s for frame #2, and 3/60 s for frame #3). Totally 8,000 gated frames (from #1 to #8,000) after UV off were identified per decay cycle to resolve the luminescent lifetime. The lifetime images were then reconstructed based on the lifetimes determined from each pixel, which was calculated by exponentially fitting the intensities of each pixel over the delay time using the gated frames. The bottom panel in Fig. 2b shows a representative smartphone lifetime image generated by Matlab, where different colors represent different lifetime values (0 to 40 s). The image clearly shows different lifetime values for the different letters. For instance, letter “C” has the shortest lifetime of around 1 s and letter “U” has the longest lifetime of about 30 s. The average pixel intensities for each of four letters were plotted (solid lines) in Fig. 2d as the luminescence decay curves, confirming the different lifetimes for different phosphors. The duration and irradiance of the UV excitation both had a strong effect on the lifetime of the luminescent materials we used to pattern the letters, and the lifetimes measured under different excitation conditions are summarized in Table S5.

To validate the lifetimes of glowing letters measured on the smartphone, we used a conventional benchtop microscope (Olympus BX43) as a comparison. Two regions of interest (ROI) were selected to be imaged by a 4× objective on the benchtop system (Fig. 2b, top, yellow and red boxes). The same pulsed LED from the smartphone device was used to excite the letters with the same irradiance of 1 mW/cm^2^ for 10 s. The benchtop microscope recorded videos at 15 fps with an exposure time of 1/60 s. The #0 frame before UV off and #1 frame after UV off are shown in Fig. 2c (top and middle panels). The lifetime images from the benchtop microscope were calculated in the same way as mentioned above, shown in Fig. 2c (bottom panels). When comparing them with the lifetime image obtained from the smartphone device, the lifetime values are quite consistent for all four letters between the different lifetime images. Moreover, the normalized intensity of luminescence decay curves generated by the two acquisition systems matched with each other well (Fig. 2d).

### The V-chopper concept

**Fig. 3.**
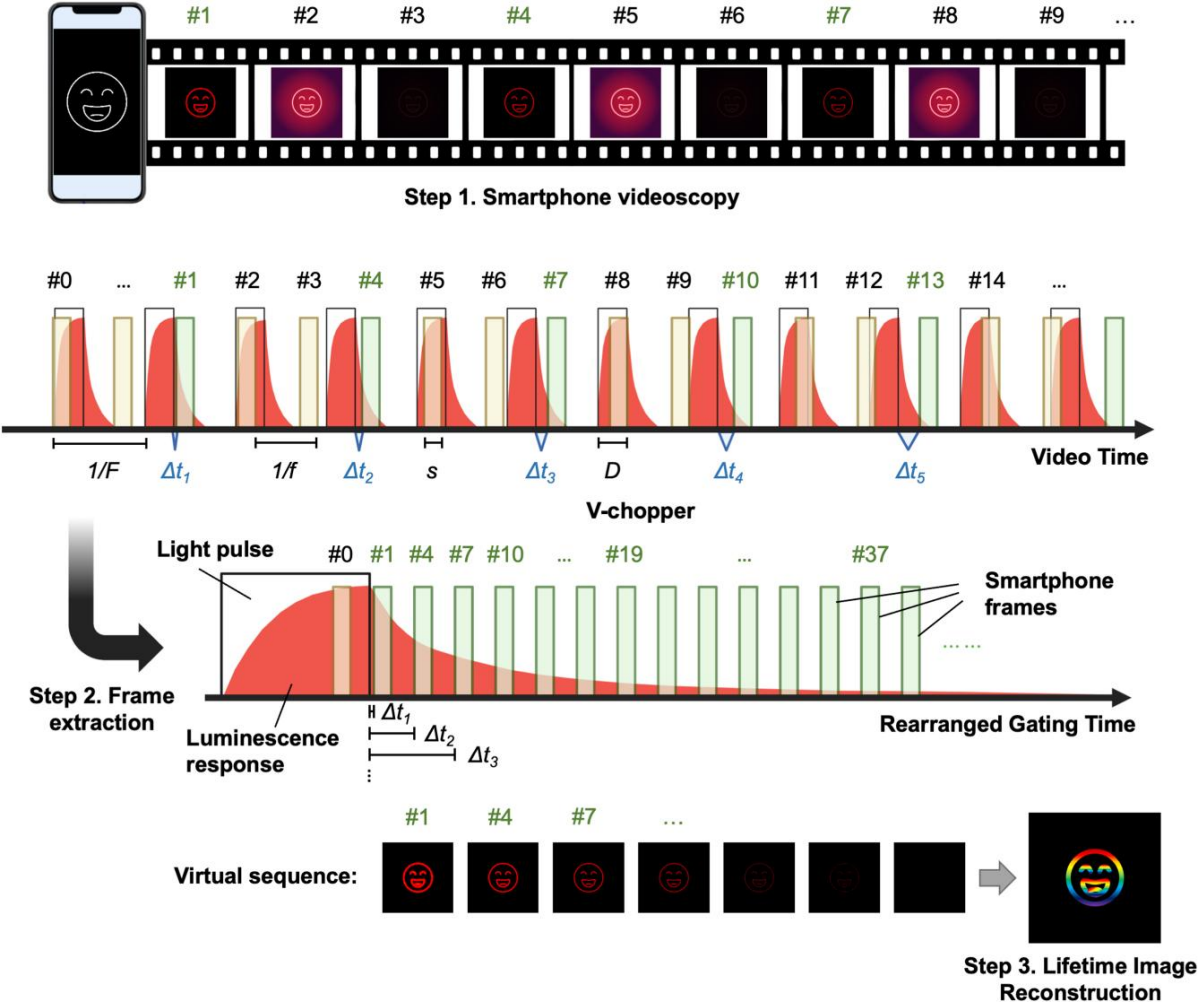
Concept of V-chopper. The method consists of three steps: (1) smartphone videoscopy, (2) frame extraction by machine learning, and (3) lifetime image reconstruction. In step 1, the large clear bars (black edge) indicate the timing when the UV excitation was on, while the translucent bars indicate the timing of the detection measurements. The red curves illustrate time-dependent emission intensity. In step 2, image frames (translucent bars) without the excitation on were collected to measure the emission lifetimes.

While it is easy to implement on the smartphone, the previous gating method (fast, multiple time gating per decay cycle) (Fig. 2) is limited by the intrinsic low frame rate of the smartphone and therefore lacks the required temporal resolution to probe faster luminescent decay events in the microsecond range. To overcome this limitation, a V-chopper method is introduced, allowing video rate smartphone device to detect microsecond lifetime signals without the need for precision excitation synchronization. The basic principle of V-chopper concept is illustrated in Fig. 3. The method consists of three simple steps: (1) smartphone videoscopy, to capture multiple cycles of luminescence decay driven by pulsed excitation; (2) frame extraction by machine learning, to isolate time-gated images (UV-off images) from different decay cycles and rearrange those frames to form a new virtual gated image sequence that can represent the illuminance decay property of the dye; and (3) lifetime image reconstruction, to calculate the lifetime value for each pixel and recover the 2D lifetime image. The V-chopper method aims to extract time-gated images from *multiple* cycles of excitation-decay events instead of a *single* decay cycle in a conventional time-gated method. The extracted gated images from different decay cycles will then be assembled to reconstruct a virtual luminescence decay curve, based on which the luminescence lifetime value will be calculated. As such, the fundamental difference between the V-chopper method and the conventional gating method is that V-chopper requires only one image gating per each or every few decay cycles, while most conventional methods rely on very fast, multiple time gating for a single decay cycle. This key difference allows us to loosen the requirement of acquisition speed (frame rate) so that a fast fluorescent event (microsecond fluorescence decay) can be captured by a low imaging rate (e.g. 30 fps) smartphone device.

### Machine learning–assisted gated image extraction

**Fig. 4.**
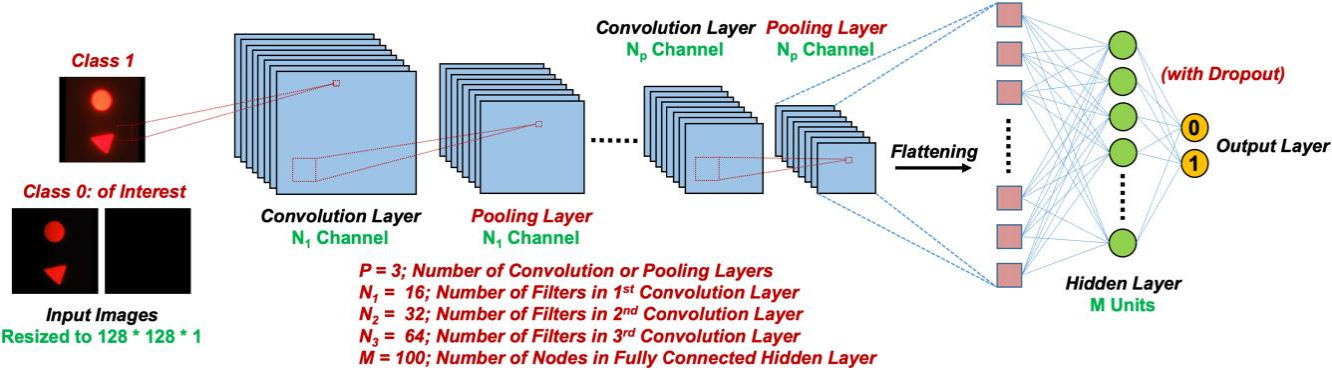
Workflow of the CNN model for automatically classifying smartphone video frames. Class 0, UV-off images, or gated frames with no autofluorescent background; class 1, UV-on images, or frames with autofluorescent background.

To streamline the selection and rearrangement of the gated images in the V-chopper method, a CNN model was designed to automatically discriminate images of interest with a high accuracy (Fig. 4). For accurate lifetime determination, the raw smartphone video frames need to be classified into two different groups: UV-on images (or class 1) and UV-off images (or class 0). After classification, all class 1 images will be discarded, and class 0 images will be rearranged based on their intensity level to form the virtual time-gated image sequence. The CNN model developed here was composed of 3 convolution layers followed by a flattened, fully connected layer including 100 hidden nodes (Fig. 4 and Table S1). Each convolution layer was succeeded by batch normalization, ReLU activation, and a max pooling layer. The fully connected layer was followed by batch normalization, ReLU activation, and dropout. Lastly, Softmax activation was utilized in the output layer to generate class probabilities, resulting in predicted labels. Additional strategies were applied to combat overfitting (see Materials and methods).

The CNN model was trained and tested with a balanced number of class 1 and class 0 smartphone images, each composed of 3,200 images. Briefly, the smartphone video frame images were converted into grayscale and resized to 128 × 128 pixels. Then, the data set was divided into three subsets—training, validation, and test sets—with a split ratio of 60/20/20. The learning process was performed in three steps: first, the CNN model was trained on the training set and applied on the validation set; second, the model was trained on both training and validation sets and applied on the test set; finally, the model was trained using the whole data set. The final trained model (after all three steps) was exported for future classification use. The trained CNN model was then applied to unknown images and separated images into two categories (class 1 or class 0). The images with predicted label “0” were the time-gated frames (UV off) which were then used to calculate the luminescence lifetime.

### Microsecond lifetime imaging by V-chopper and machine learning

**Fig. 5.**
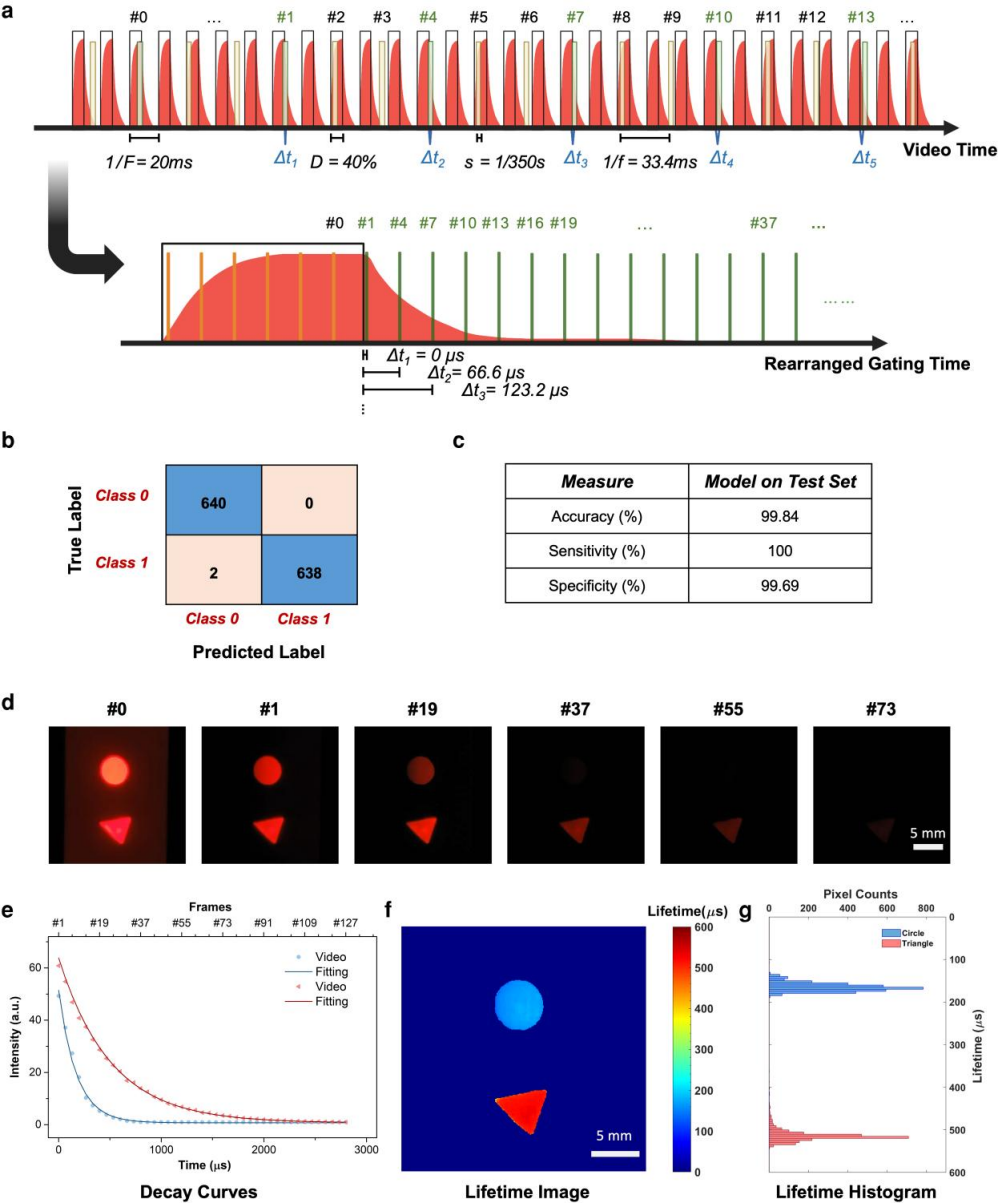
Demonstration of the smartphone V-chopper method for microsecond lifetime imaging. a) Schematic of the smartphone video sequence and pulsed excitation timelines before and after frame extraction and gating image rearrangement. b) Confusion matrix of the CNN model. c) Performance of the CNN model trained by the combined training/validation data sets and applied on the test set. d) Representative #0 frame and gated frames extracted from the smartphone video by the CNN model. e) The decay curves of circle (blue) and triangle (red) over gated time, f) the reconstructed lifetime image of the microsecond probes, and g) the corresponding lifetime histograms from the two pattern spots.

For a proof-of-concept demonstration of the V-chopper method, two Eu probes with distinct lifetimes in the range of microseconds were patterned on a paper substrate in different shapes (Fig. 5 and Materials and methods). One Eu chelate, 4,4′-bis(1″,1″,1″,2″,2″,3″,3″-heptafluoro-4″,6″-hexanedion-6″-yl)-chlorosulfo-o-terphenyl-Eu^3+^ (BHHCT-Eu^3+^) with a shorter lifetime (∼250 µs), was patterned in the round shape, and Eu microbeads with a longer lifetime (∼500 µs) were dispersed in a triangle shape. The sample slide was illuminated using the UV LED at a pulse frequency of 50 Hz and 40% duty cycle, and the phosphorescence was recorded at 30 fps and 1/350 s exposure time set on the smartphone (Fig. 5a and Videos S2 and S3). A series of time-gated images from different modulation cycles were generated (Fig. S1b). The frame with UV on is assigned the #0 frame, and the first frame after the UV is off is referred as the #1 frame, as usual. The time delay of #1 frame is close to 0 ($\Delta t_{1}∼0$). Subsequently, #4, 7, 10, … are time-gated images identified by the CNN model with different decay times $\Delta t_{n}$. Although the preset frame rate was 30 fps, we noticed the actual frame rate was around 29.98 fps. The little shift of the smartphone video rate is actually critical to generate small time delays without the need for expensive control devices. The interplay between video rate and excitation frequency was explored in more detail by a modeling method as described in the Discussion section. Finally, according to Eq. 6, time interval Δt is 22.2 µs between two successive frames for an actual frame rate of 29.98 fps. For a UV pulse of 50 Hz, one gated image was identified for every three consecutive image frames, and therefore, the gated interval is $\Delta t_{2}=66.6\;\;\mu s,\;\Delta t_{3}=123.2\;\;\mu s$, and so on for the rearranged virtual image sequence.

The developed CNN model classifies time-gated images with a high accuracy. Figure 5b and c shows the confusion matrix as well as the performance measures of the CNN model. The CNN model trained on the combination of training/validation sets was found to perform well with a training and test accuracy of 99.90 and 99.84%, respectively (Fig. 5c). The CNN model trained on the whole data set was subsequently applied to 5,317 unseen images to classify. The smartphone images were automatically separated into two different categories by their predicted labels. The representative #0 frame and CNN-classified time-gated images are shown in Fig. 5d. The lifetimes of these two dyes can be easily distinguished in the gated frames. The circle is barely visible after frame #37, while the triangle still glows slightly in frame #73, indicating the much longer lifetime of triangle (Eu microbeads) than the circle (BHHCT-Eu). The average pixel intensities of the circle (blue) and triangle (red) were plotted as a function of time (Fig. 5e). When fitted with the monoexponential decay curves, the lifetime of the circle was calculated to be 168.4 µs, and the lifetime of the triangle was 512.3 µs. A reconstructed lifetime image was generated by fitting the intensity of each pixel over time and calculating the lifetime for each pixel (Fig. 5f). A clear difference in the lifetimes of the circle and the triangle was visualized based on the color bar. The corresponding lifetime histogram of each pattern is shown in Fig. 5g, showing a narrow distribution of ±9.3 µs (coefficient of variation 5.5%) and ±14.5 µs (coefficient of variation 2.8%) for the circle and the triangle, respectively. Both lifetimes determined on the smartphone V-chopper device were highly consistent with the results measured on the commercial time-resolved spectrometer, which were 167.2 ± 1.4 µs and 516.4 ± 7.0 µs for the circle and the triangle, respectively (Fig. S2 and Table S2). The error percent is within 0.8% when comparing the smartphone V-chopper and benchtop spectrometer results.

**Fig. 6.**
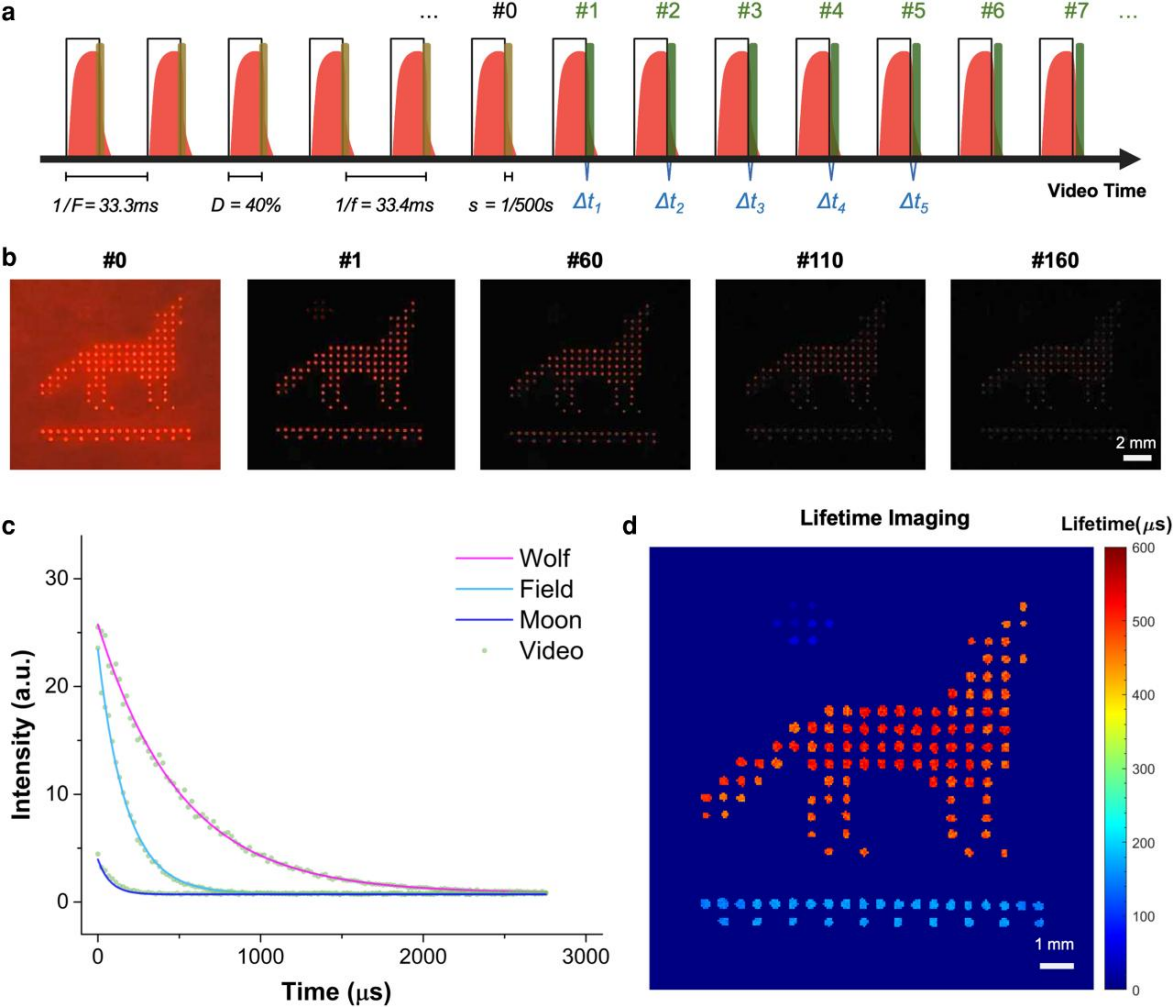
Detection of subhundred microsecond lifetime on the smartphone V-chopper device. a) Schematic of the smartphone video sequence and pulsed excitation settings. b) Representative gated frames extracted from the smartphone video by the CNN algorithm. c) The decay curves of luminescent intensities over gated time. Green dots are actual experimental data, and solid lines are exponential fitting curves. d) The reconstructed lifetime image of the time-encoded pattern. The shortest lifetime pattern (moon) is clearly visualized.

To demonstrate the detection of subhundred microsecond lifetime on the smartphone V-chopper system, three different luminescent dyes were microprinted in a picture of a howling wolf (Fig. 6). The wolf, ground, and moon consisted of Eu microbeads (lifetime ∼500 µs), BHHCT-Eu chelate (lifetime ∼250 µs), and tetracycline hydrochloride (Tc) Eu dye (Tc-Eu) (lifetime <100 µs), respectively. The smartphone took videos at 30 fps (29.98 fps in real data set) with 1/500 s exposure time, and the UV LED was pulsed at a frequency of 30 Hz with a 40% duty cycle. That we are able to perform this experiment by using the same rate for both video recording (30 fps) and excitation pulse (30 Hz) is again due to the fact that the actual video rate (∼29.98 fps) always deviates from the nominal value a little bit. Due to this mismatch, when a long video was recorded, the image frames gradually shifted away from the UV pulses, generating gated UV-off images (Fig. 6a and Videos S4). Then, the gated frames in successive cycles were gathered to form the virtual decay image sequence. The delay time $\Delta t_{n}$ was measured as 0 µs, 22.2 µs, 44.4 µs, and 66.6 µs, respectively, for gated frame #1, #2, #3, and #4, making it highly possible to resolve lifetime between 50 and 100 µs. This setting requires a long video to be recorded to allow the frames to scan over the whole decay curves, especially when the lifetime is over hundreds of microseconds. The minimum video duration (Eq. 8) for efficient lifetime resolving is considered further in the Discussion section.

Figure 6b displays frame #0 (UV on), frame #1 (the first gated frame after UV off), frame #60, and so on. Comparing the gated frames #1-160, it is obvious that the phosphor in the wolf is the most long-lived and the phosphor in the moon is the shortest-lived—it is barely visible after frame #3. The decay curves of three dyes extracted from all gated frames were plotted in Fig. 6c (green dots) and fitted by exponential functions (solid lines) to resolve the lifetimes. The recovered lifetime image (Fig. 6d) shows three distinct lifetime values (510.4 µs, 169.3 µs, and ∼78.3 µs) for the wolf, ground, and moon, respectively, corresponding to the advertised lifetime of each luminescent material (Fig. S2 and Table S2). The data suggest that by applying the V-chopper concept, short lifetimes below 100 µs can be resolved on a portable smartphone reader device with a relatively low frame rate at 30 fps.

## Discussion

A conventional time-resolved luminescence detection system is usually composed of a light source capable of pulsed excitation, a gated optical detector to capture time-dependent luminescence, and a synchronized control unit to provide the phase difference (or time delay) between the excitation and detection windows. Nowadays, laser diodes and LEDs can achieve terrific temporal resolutions with repetition rates up to 100 MHz and pulse width of ns to µs while maintaining cost-effectiveness and portability. Therefore, the gated optical detector becomes a key challenge in the implementation of time-gated detection, especially for a portable time-resolving system which needs a very high acquisition rate for multiple samplings in each cycle to fit the luminescence decay exponentially.

On the other hand, over the past decades, the smartphone has been widely explored as a capable analytical sensing and imaging platform in many POC applications, such as disease diagnostics, environmental monitoring, and screening for food contamination (41–46). In most of the previous applications, smartphone cameras are used to take individual photos for data analysis. Recently, with the rapid technological advancement of complementary metal oxide semiconductor (CMOS) cameras equipped on the smartphone, users can have more control of the video capturing mode such as the exposure time, frame rate, and focusing distance. In the recent half decade, many smartphone models on the market have achieved super high frame rate videos (above 100 fps) with high resolution definition and opened an emerging analytical method defined as smartphone videoscopy (30).

However, previously reported smartphone-based lifetime quantification methods still require complicated mechanical chopper systems to achieve high temporal resolution, limiting the time-resolved technologies for POC use (Table S4) (35–37). Here, instead of using expensive high-speed image sensors or complicated mechanical choppers, we demonstrated a virtual modulation method, the V-chopper, by generating controlled time shift between video frames and light pulses to reconstruct luminescence decay curves from multiple modulation cycles. The gated image extraction and rearrangement are fully automated by a CNN deep learning model. By applying the V-chopper method, the lifetime as short as a few tens of microseconds can be resolved on a consumer smartphone device using a low video frame rate (e.g. 30 fps). Compared with previous mechanical chopper-based systems, the smartphone V-chopper platform is much more cost-effective and easier to implement. Meanwhile, the concept of the V-chopper can be broadly applied not only to smartphone detectors but also conventional digital cameras or image sensors. The latter can provide more precise control of frame rate and exposure time and has the potential to even resolve nanosecond lifetime.

To perform smartphone V-chopper, it is important to select a good combination of excitation and video recording settings. A Matlab-based simulation program (Fig. S3 and codes available at https://github.com/VictoriaYanWang/Smartphone-Lifetime-Imaging) was developed to study the interplay of the light pulse frequency (*F*), duty cycle (*D*), video recording rate (*f*), and shutter speed (*s*). The following general rules should be followed for a successful V-chopper implementation on the smartphone.

### Excitation pulse

To detect the luminescence lifetime (*τ*) precisely, the requirement for the LED pulses is that the LED off time (defined by (1−D)/F) should be longer than the whole decay curve or longer than at least 3 times the lifetime *τ*. In other words, the next excitation should not be turned on until the previous decay curve is completed.


(1−D)/F≥3⋅τ. (1)

For instance, if the LED duty cycle is set at 40%, then the LED pulse frequency (*F*) should meet the following condition:


$$F\leq \frac{1}{5\tau }\;.$$ (2)

Therefore, based on the estimated lifetime of the dye to be detected, the repetition frequency of LED can also be selected accordingly. For example, for dyes with 10 ms lifetime, excitation with 20 Hz or lower frequencies (40% duty) should be used (Table S3). In contrast, for ultralong lifetimes, excitation with 1 Hz or lower should be applied to match the long decay curve.

### Shutter speed

Conditions for a successful time-resolved detection also involve the setting of shutter speed (*s*), which should be shorter than the LED off time; otherwise, the camera will always capture LED on images. The condition can be expressed as


s<(1−D)/F. (3)

For example, for LED frequency of 20 Hz and 40% duty cycle, the shutter speed should be shorter than 0.03 or 1/33 s (Table S3).

### Frame rate

The choice of frame rate on most smartphones is very limited. The values are discrete instead of being successively adjustable. However, the few options of preset frame rate will not limit the capacity of smartphone V-chopper device to resolve a broad range of lifetimes, including the short lifetimes in the microseconds. It is worth mentioning there is sometimes a small drift to the preset frame rate when a video is taken on the smartphone. Based on the information stored in the video properties, the actual frame rate *f*_real_ may be slightly different from the preset values (e.g. *f* = 24, 30, or 60 fps) with a tiny drift *σ* (0.01 to 0.30 Hz). For the current phone model we use, the *f*_real_ is often 30.02 or 29.98 fps for a given preset rate of 30 fps.


$$f_{\mathrm{real}}=f\pm \sigma .$$ (4)

The small drift of the smartphone frame rate provides a simple phase shifting mechanism between video frames and LED pulses, which provides the shortest delay time of gated frames that we can achieve in the current setup for measuring short luminescence lifetimes.

If the smartphone videoscopy is set at 30.00 fps sharp with 1/350 s exposure time and LED has 50 Hz frequency with 40% duty cycle, the intensities of gated frames will be the same across the whole video since the frame window sits at the same delay time for each luminescence decay ($\Delta t_{n}$ is a constant; Fig. S4a). However, for a frame rate of 29.98 fps (Fig. S4b) or 30.02 fps (Fig. S4c), the gated frames started to show modulated intensities across frames. That is because the small drift provides a phase shift to video frames which modulates $\Delta t_{n}$ over the decay curves. The varied $\Delta t_{n}$ allows video frames to sample different parts of the luminescence decay curves in multiple time-gated cycles. More specifically, if $\;f_{\mathrm{real}}<f$, the intensity of the gated frames decreases as a function of time, meaning that gated frames scan the luminescence decay curve from high to low intensity (or shifting away from the LED pulses). In contrast, if $\;f_{\mathrm{real}}>f$, the gated frames are shifting toward the LED pulses, so the intensity of the gated frames increases accordingly.

The delay time between successive frames Δt will be equal to the drifting time between the actual frame rate and preset frame rate, which can be defined by


$$\Delta t=\left|\frac{1}{f}-\frac{1}{f_{\mathrm{real}}}\right|=\frac{\sigma }{f\cdot f_{\mathrm{real}}}.$$ (5)

Therefore, the delay time between two successive gated frames $\Delta t_{n}$ will depend on frame numbers *m* between each two, which means


$$\Delta t_{n}=(n-1)\cdot m\cdot \Delta t.$$ (6)

And *m* can be calculated by


m=f/gcd(f,F), (7)

where gcd represents the greatest common divisor. For example, for a frame rate of 30 fps and UV pulse of 50 Hz (40% duty), m=3; therefore, $\Delta t_{n}=3(n-1)\cdot \Delta t$, and $\Delta t_{1}$ is always close to 0. According to Eq. 5, Δt is 11 µs for f=30fps and σ=0.01fps, and Δt will be 2.78 µs when f=60fps and same *σ*. It is clear that the smaller frame drift and higher frame rate that the smartphone camera can provide, the smaller time delay Δt would be. The minimum delay time can predict the limit of detection (LOD) for lifetime by the smartphone V-chopper device on the order of tens of microseconds, which is equivalent to the results obtained by the previous smartphone systems equipped with mechanical choppers and motor for lifetime detection (35–37).

Based on the above general rules and Eqs. 1–6, the recommended settings for successful smartphone V-chopper implementation on the different lifetime ranges can be found in Table S3.

### Video duration

The minimum duration of a video clip ($T_{\min}$) to capture in order to scan over a whole decay curve for lifetime detection follows the equation:


$$T_{\min}=\frac{(1-D)/F}{\Delta t}\cdot \frac{1}{f_{\mathrm{real}}}=\frac{(1-D)/F}{\sigma /f}=\frac{f}{\sigma \cdot F}\cdot (1-D).$$ (8)

According to Eq. 8, the minimum necessary length of a video recorded to resolve the lifetime is proportional to the video frame rate (*f*) and inversely proportional to the LED pulse frequency (*F*). As such, a lower video frame rate combined with higher frequency LED pulses is more time-efficient for lifetime detection on the smartphone.

Many smartphone CMOS sensors are controlled by the ERS, which can lead to different readout times of each line of the gated image and therefore affects the accuracy of lifetime calculation. We compared three different smartphone models (iPhone 13 Pro, LG V10, and Samsung Galaxy S9) and evaluated the effect of ERS on V-chopper applications (Figs. S8–S11). The results showed that Samsung S9 had the minimum ERS effect probably due to their ERS effect cancellation technology by using a unique three-layer stack image sensor construction. In addition, several ERS compensation algorithms (47, 48) can be applied to further reduce the ERS effect if needed.

In this work, we focus on the methodology and the proof-of-concept of the V-chopper system. Follow-up studies to demonstrate real applications for sensitive biomarker detection are undergoing. Moreover, the concentrations of Eu dye in the current experiments are relatively high (0.01 to 0.1 M) compared with those used in the real applications, which are normally below 1 mM. It will be challenging to image luminescent molecules at low concentrations by the current smartphone setup without any external objective lens and signal amplification technologies. In future, a more powerful excitation source like pulsed laser and objective lens could be introduced to further improve the optical sensitivity of the system and therefore unlock the full potential for biosensing.

In summary, a low-cost smartphone-based lifetime imaging platform has been developed for time-gated detection and 2D lifetime imaging over a broad range of lifetime from microseconds to seconds. To probe low microsecond lifetime events, a V-chopper method was demonstrated by modulating the LED pulses and smartphone video frame rate accordingly. Coupled with machine learning for gated image extraction, the V-chopper method offers opportunities to resolve fast luminescence decay events on a low frame rate image sensor. The minimum lifetime that can be detected by the smartphone V-chopper system is about 75 μs, which is comparable with or even lower than that obtained from previous mechanical chopper-based smartphone systems. This V-chopper method decouples the traditional time-resolved detection from expensive and complicated instruments. The miniaturized smartphone V-chopper system exhibits huge potentiality in lifetime imaging for various applications such as POC biosensing. The methodology can also be a universal method, which can be applied to benchtop sensors to resolve even faster fluorescence events in the future.

### Online content

Any methods, additional references, Nature Portfolio reporting summaries, source data, extended data, supplementary information, acknowledgements, peer review information, details of author contributions and competing interests, and statements of data and code availability are available at https://doi.org/.

## Materials and methods

### Preparation of the smartphone V-chopper device

The smartphone V-chopper lifetime imaging prototype device consists of a 3D-printed enclosure, a UV LED (365 nm, M365L3, Thorlabs), a condenser lens (ACL2520U-A, Thorlabs), a UV-enhanced reflection mirror (PFSQ10-03-F01, Thorlabs), and a smartphone (Samsung Galaxy S9). The sample glass slides can be placed inside the enclosure on the bottom. The UV LED is controlled by a LED driver (LEDD1B, Thorlabs) and pulsed via a square wave voltage source (DG1062Z, Rigol). The highly divergent emission from the UV LED was first collimated by the aspheric condenser lens (*f* = 20.1 mm, NA = 0.60) and then evenly projected on the glass slide by a tilted reflection mirror (Fig. 1a). The tilted angle of the 1“ × 1” UV-enhanced mirror is designed to be ∼67.5 degree relative to the slide surface, so the LED can deliver a uniform illumination to the sample slide, and meanwhile, the illumination center is aligned with the field of view (35 × 63 mm^2^) of the smartphone. When detecting luminescent signals from Eu complex dyes, a 615 nm band-pass filter (87-739, Edmund Optics) can be mounted in front of the phone camera to eliminate excitation interference. The Galaxy S9 smartphone has manual control of video settings, e.g. ISO, focal length, shutter speed, video frame rate, and image resolutions. A 60 fps video frame rate was used for lifetime detection of ultralong luminescent materials (seconds). For the measurement of microsecond lifetime targets, a normal video rate (30 fps) was used instead combined with the V-chopper principle.

### Preparation of Eu dyes

The long lifetime luminescence probes are often lanthanide-based complexes and nanoparticles, with luminescence lifetimes typically in the range of 1 μs to 10 ms. Ultralong or persistent lifetimes can last for a few seconds or even up to minutes. Here, Eu probes with different labeled lifetimes in microseconds, milliseconds, and seconds range have been used for demonstration of lifetime imaging with V-chopper on the smartphone videoscopy. To demonstrate the concept of using V-chopper for time-resolving detection and lifetime imaging, the dyes described above have been coated on paper substrates, which have strong autofluorescence to present as background noise. The ultralong lifetime powders (micron size particles) with different colors and glow durations (shown in Table S2, Techno Glow) have been selected to demonstrate resolving lifetimes over hundreds of milliseconds and up to seconds. The red glowing powder contains calcium sulfide, and the other three powders have composition of strontium aluminate europium dysprosium (SrAl_2_O_4_:Eu^2+^, Dy^3+^). To prepare different patterns, sticker labels were cut into “N,” “C,” “S,” and “U” shapes to trap glow powders evenly on the adhesive side. Then, four letters with different colored powders were placed on an autofluorescent paper substrate which was then sandwiched with two glass slides. Excess Tc powder (T2525, TCI) was dissolved in 0.1 M Na_2_CO_3_ butter (pH = 8). Then, EuCl_3_ solution was added to generate Tc-Eu chelate. Filter paper (09801B, Fisherbrand) was then soaked in the Tc-Eu dye suspension and measured immediately when wet. BHHTC-Eu (59752, Sigma-Aldrich) with bright emission under UV was dissolved in DMSO with a concentration of 0.01 M to coat the filter paper. The original suspension of 0.2 µm Eu chelate polystyrene beads (S9347, Thermo Fisher) was diluted by 100 times with MilliQ water and then spiked on the filter paper.

The Eu chelate dye Tc-Eu, BHHCT-Eu, and Eu microbeads in Table S2 have been calibrated with a commercial time-resolved spectrometer (LP920, Edinburgh Instruments). The emission spectra peak of the Eu chelates is at ∼615 nm when excited at the UV 365 nm (Fig. S5). The emission spectra of paper substrate and substrate with Eu dye were demonstrated in Fig. S6. The red spectra (solid and dash) were measured when UV was on, and the blue ones had a 10 µs delay time after UV exposure. It shows the paper substrate has strong autofluorescence under UV (red dash), which can be eliminated with a 10 µs gate time (blue dash). However, the long-lived Eu dye is still luminescent, peaking at ∼615 nm (blue solid). The lifetimes of Eu dyes have been measured by the time-resolved spectrometer as shown in Table S2 and Fig. S2.

### Simulation of the V-chopper mechanism

To visualize the time delay (Δt) in the V-chopper method and study the interplay of the light pulse frequency, duty cycle, video recording rate, and shutter speed, a simulation program with user interface was designed in Matlab. The program is now available to download on GitHub (https://github.com/VictoriaYanWang/Smartphone-Lifetime-Imaging), which will help users find an optimal excitation and data acquisition setting for a given lifetime target. When the LED pulse frequency (Hz), duty cycle (%), smartphone frame rate (fps), shutter speed (*s*), and estimated lifetime of the target dye are input in the interface window, two tracks of pulses will be generated, namely the waveform of LED (Fig. S3b, blue solid line) and waveform of smartphone frames (Fig. S3b, green solid line). Each smartphone frame will be assigned a frame number (e.g. #1, #2, and #3), shown on top of the video frames. In addition, the decay curves of luminescence (Fig. S3b, red dash line) will also be simulated following each LED excitation pulse. The X-axis represents time (s), and the Y-axis with arbitrary units represents intensity for luminescence. The example simulation result shown in Fig. S3b demonstrates when LED has a repetition rate of 50 Hz and duty cycle of 40%, and the frame rate of smartphone video is 30.02 fps with 1/350 s shutter speed. The gated frames will be found only where the decay curve (Fig. S3b, red dashed line) and smartphone frames (Fig. S3b, green solid line) are overlapped, marked in magenta (Fig. S3b).

The size of the magenta area corresponds to the amount of light being collected in this frame, which is proportional to the luminescence intensity of the gated frames. Therefore, the luminescent intensity of each frame can be calculated by accumulating the area of magenta segments and plotted over time (Fig. S7a). Several exponential curves (decay *n*, *n* + 1, *n* + 2…) can be synthesized in a 30 s observation window. Among these virtual decay curves, decay *n* + 2 is a complete curve that can be used for accurate lifetime calculation. Figure S7b shows the actual frames extracted from the video which was taken with the settings used in Fig. S3b.

### The CNN classification model

The basic structure of the CNN model is illustrated in Fig. 4. To combat overfitting, several techniques were applied simultaneously: (i) batch normalization was utilized after each convolution and the fully connected layers, (ii) dropout was employed after the fully connected layer, and (iii) early stopping was used to stop the training process when the validation loss reaches its minima. The RMSprop optimizer with the learning rate of 0.0001 was adopted to compute the model weights and biases.

For training, the smartphone image data set was prepared with the balanced classes (0 or 1), each composed of 3,200 images. A 60/20/20 split was utilized to initiate training, validation, and test sets, respectively. The performance of the CNN models was assessed using accuracy, sensitivity, and specificity that could be calculated based on the following equations:


Accuracy=(TP+TN)/(TP+TN+FP+FN) (9)

Sensitivity=TP/(TP+FN) (10)

Specificity=TN/(TN+FP) (11)

where TP is the true positive (the number of class 0 images classified correctly), TN is the true negative (the number of class 1 images classified correctly), FP is the false positive (the number of class 1 images the model classifies incorrectly as class 0), and FN is the false negative (the number of class 0 images the model classifies incorrectly as class 1).

After training, a user-friendly code was written to apply the trained CNN model to classify unseen smartphone images. The Python code for training the CNN model, the model application code, and the smartphone images used as the data set to develop and evaluate the model are freely available on GitHub (https://github.com/VictoriaYanWang/Smartphone-Lifetime-Imaging).

## Supplementary Material

pgad313_Supplementary_Data

## Data Availability

The authors confirm that the data supporting the findings of this study are available within the article and its Supplementary materials. The Python code for training the CNN model, the model application code, and the smartphone images used as the data set to develop and evaluate the model are freely available on GitHub (https://github.com/VictoriaYanWang/Smartphone-Lifetime-Imaging).
