# Supplementary material for: Low-rate smartphone videoscopy for microsecond luminescence lifetime imaging with machine learning
Source: PNAS Nexus. 2023 Sep 27;2(10):pgad313. doi: 10.1093/pnasnexus/pgad313 (PMC10566544; doi:10.1093/pnasnexus/pgad313)
Supplement: pgad313_Supplementary_Data [file pgad313_supplementary_data.zip › PNASNEXUS-PNASNEXUS-2023-00496R-s05.docx]

**
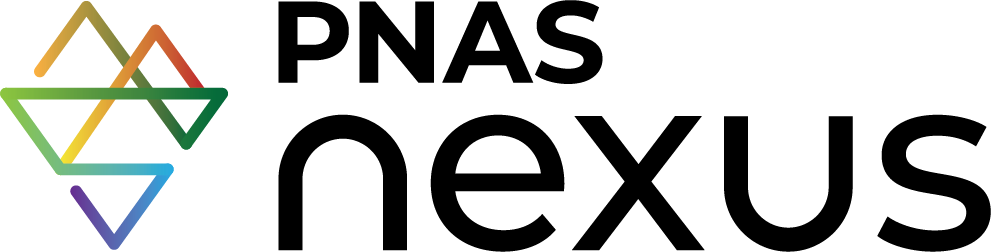
**

**Supplementary Information for**

Low-Rate Smartphone Videoscopy for Microsecond Luminescence Lifetime Imaging with Machine Learning

Yan Wang^1^, Sina Sadeghi^1^, Alireza Velayati^1^, Rajesh Paul^1^, Zach Hetzler^1^, Evgeny Danilov^2^, Frances S. Ligler^3^, Qingshan Wei^1^*

^1^ Department of Chemical and Biomolecular Engineering, North Carolina State University, Raleigh, NC, 27695 USA.

^2^ Department of Chemistry, North Carolina State University, Raleigh, NC, 27695 USA.

^3^ Department of Biomedical Engineering, Texas A&M University, College Station, TX 77843, USA.

* Qingshan Wei

**Email:** [qwei3@ncsu.edu](mailto:qwei3@ncsu.edu)

**This PDF file includes:**

Figures S1 to S11

Tables S1 to S5

Legends for Videos V1 to V4

**Other supplementary materials for this manuscript include the following:**

Videos V1 to V4

**
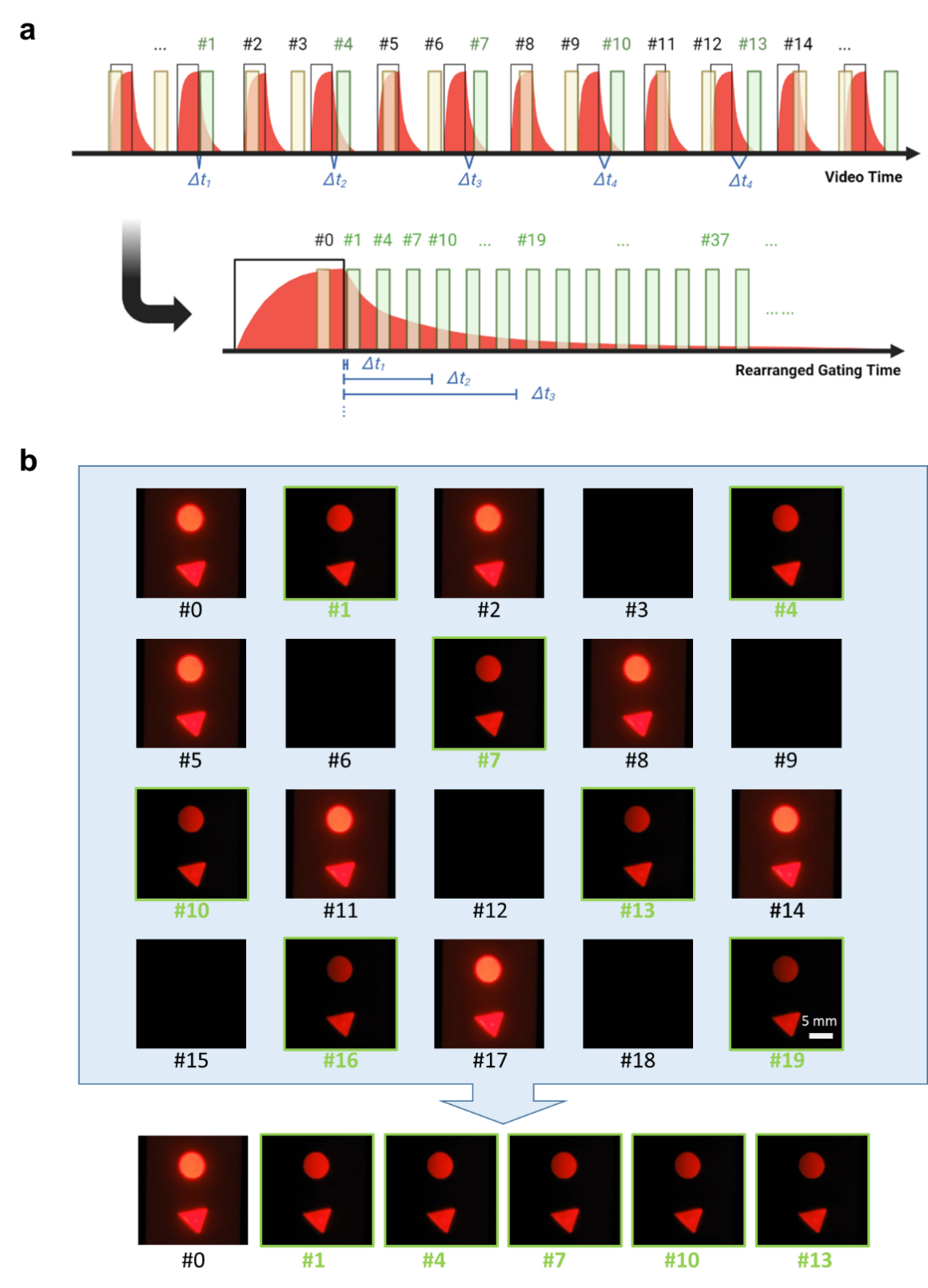
**

**Fig. S1. Raw video frames from the smartphone for microsecond lifetime imaging. a** Schematic of the smartphone video sequence and pulsed excitation timelines before and after frame extraction and gated images rearrangement. In the upper row, the large clear bars (black edge) indicate the timing when the UV excitation was on, while the translucent bars indicate the timing of the detection measurements. The red curves reflect time-dependent emission intensity. In the lower row, data collected from frames (translucent bars) without the excitation on were collected to measure the emission lifetimes. **b** The gated images was classified from the original extracted frames by applying the CNN model. **
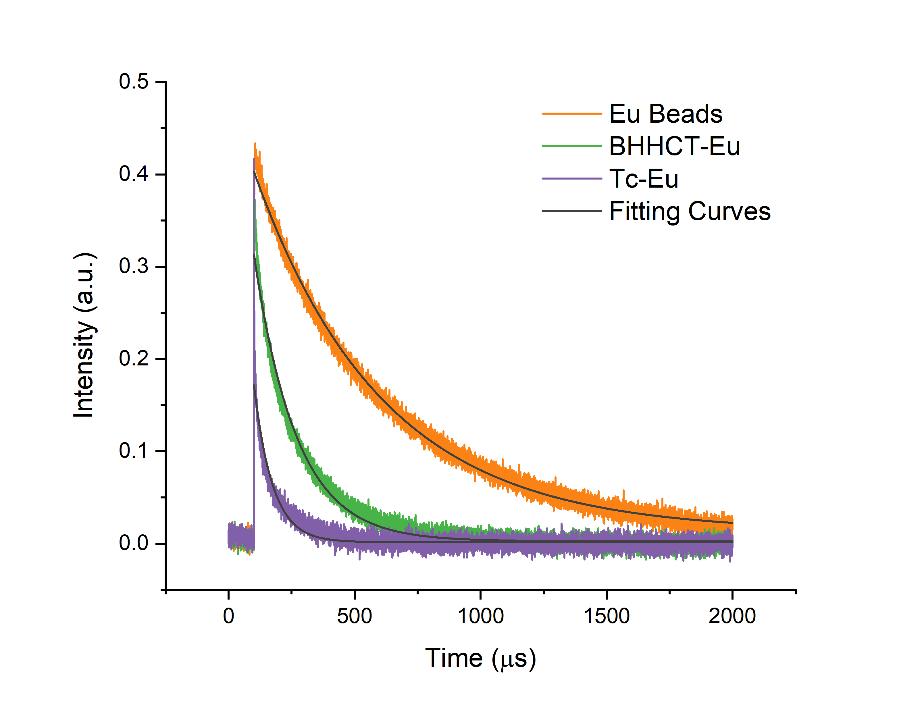
**

**Fig. S2. Calibrated luminescent decay curves of three Eu dyes used in this paper.** A commercial benchtop time-resolved spectrometer (LP920, Edinburgh Instruments) was used to calibrate the lifetimes, which have been demonstrated in **Table S2**.


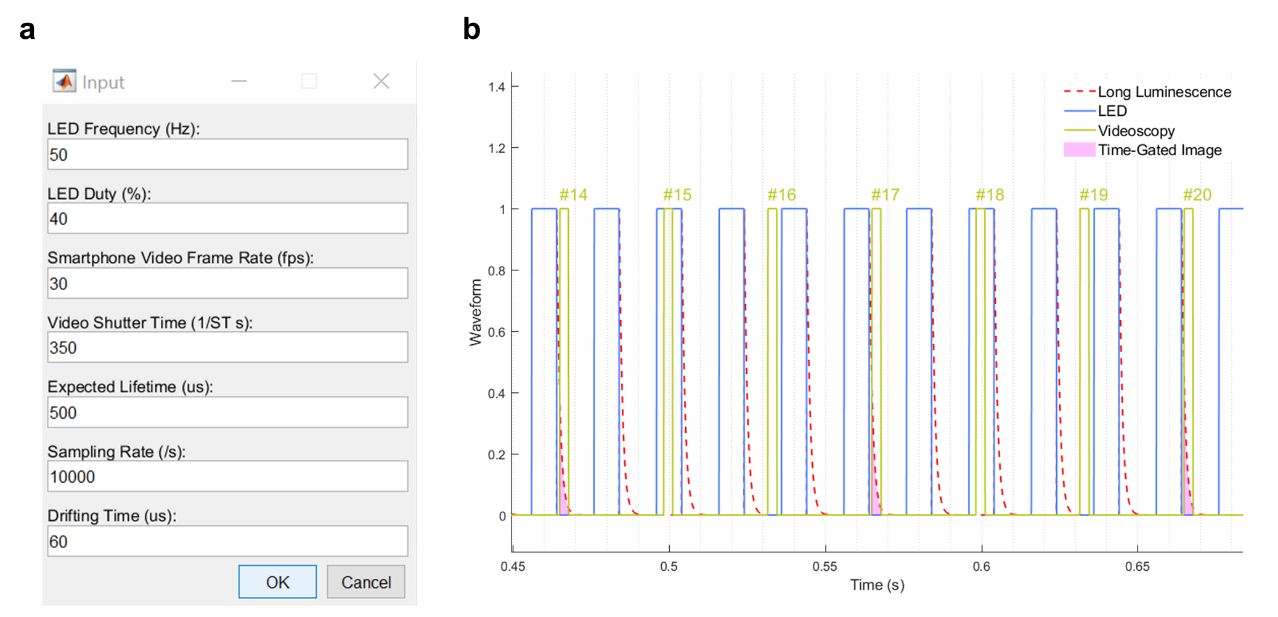


**Fig. S3.** **Matlab GUI for smartphone videoscopy simulation. a** Input window. **b** Signal train of simulated LED pulses (blue solid), V-chopper (green solid) and decay of long luminescence (red dash). The overlap area of V-chopper and luminescence represents the intensity of gated frames captured in the sequence (magenta segments), which have been plotted in **Fig. S4**.


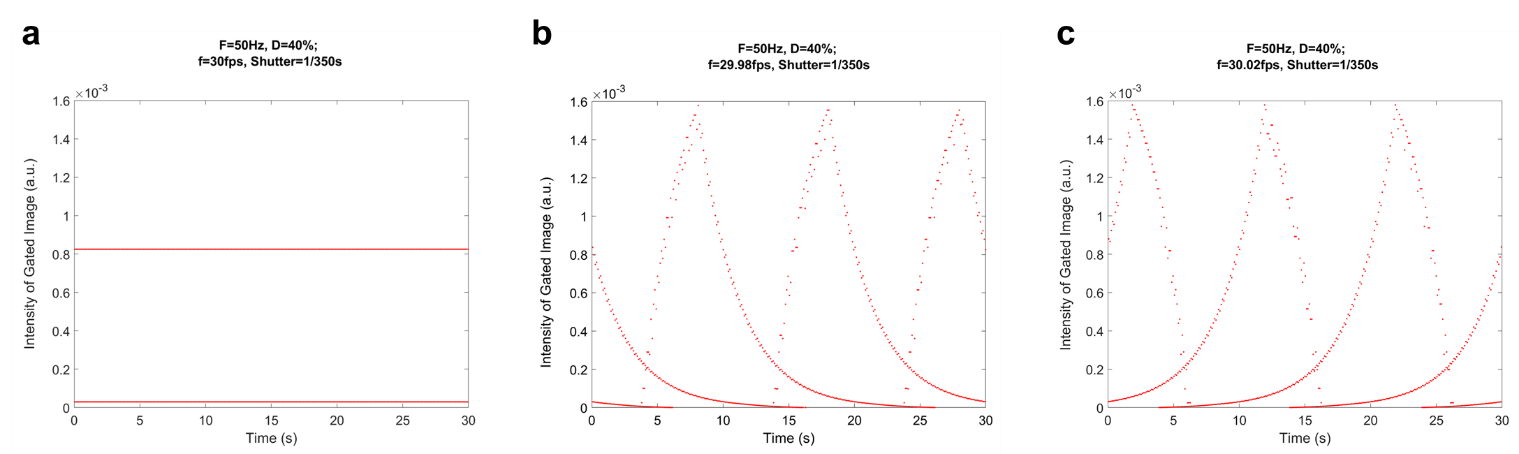


**Fig. S4. Simulated intensity of gating frames in a 30 seconds video when recorded with (b and c) or without (a) drifting to preset 30 fps frame rate. a** The intensities of gated frames are the same across the whole video when frame rate is sharp 30 fps ($\Delta t_{n}$ is a constant). **b and c** The gated frames started to show modulated intensities across frames, and **b** shows decreased intensity modulation when $f_{real}<f$, and **c** shows increased modulation if $f_{real}>f$.

**
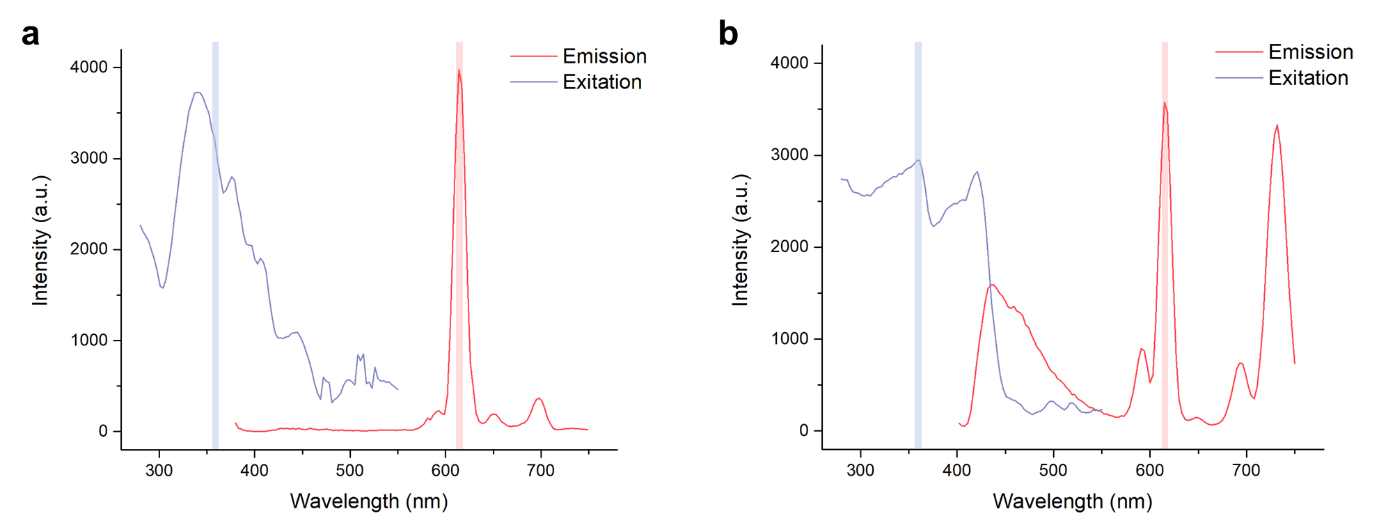
**

**Fig. S5. Excitation and emission spectra of Eu dyes. a** Eu microbeads spectrum measured in a water suspension. **b** Tc-Eu dye spectrum measured in 0.1 M Na_2_CO_3_ butter (pH=8).

**
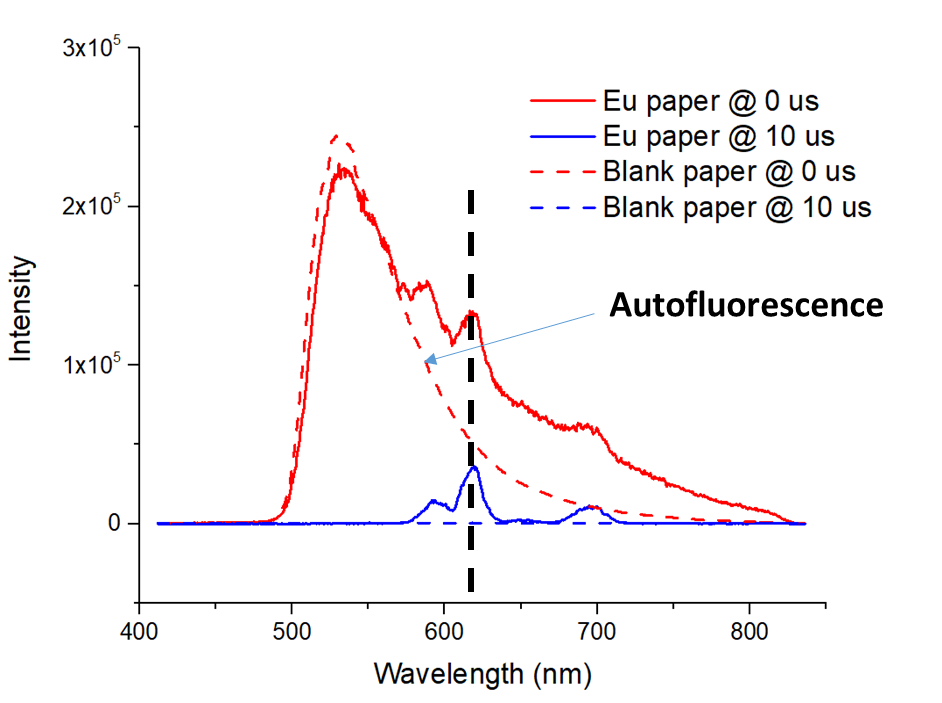
**

**Fig. S6. The emission spectra of blank paper substrate and substrate soaked with Eu dye.**


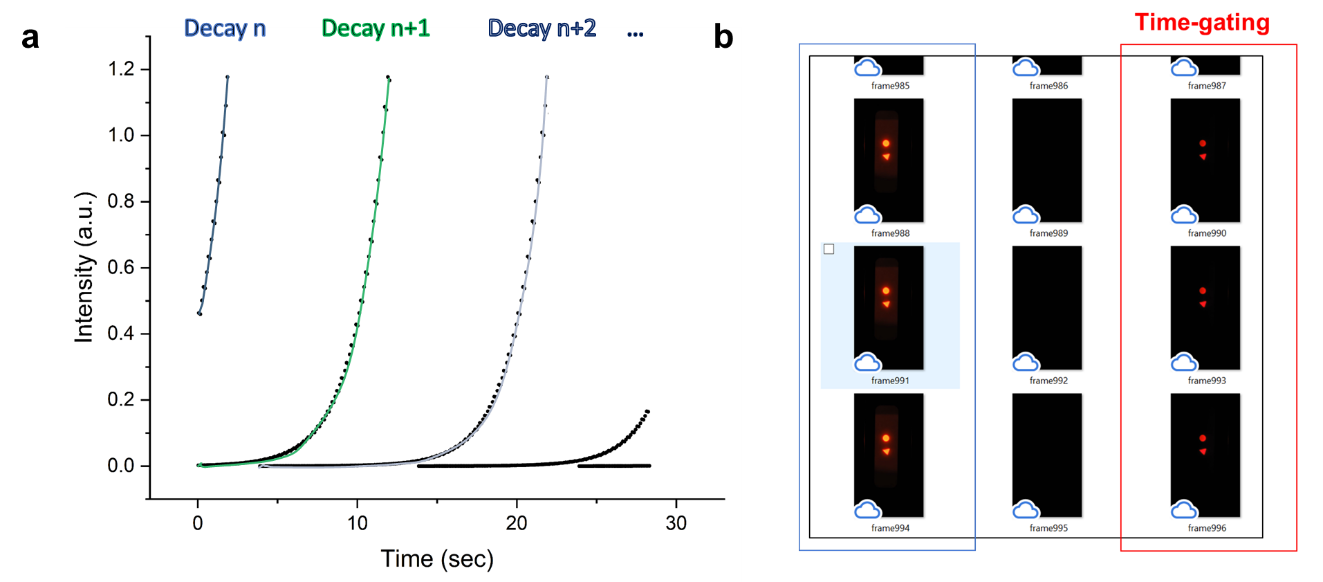


**Fig. S7. a** Luminescence intensity of gated frames calculated based on the simulated results. In a 30 seconds video clip, the whole decay of long-lived luminescence signal has been fully captured in the Decay n+2. **b** Actual gated frames that mixed with autofluorescent frames in the original video.


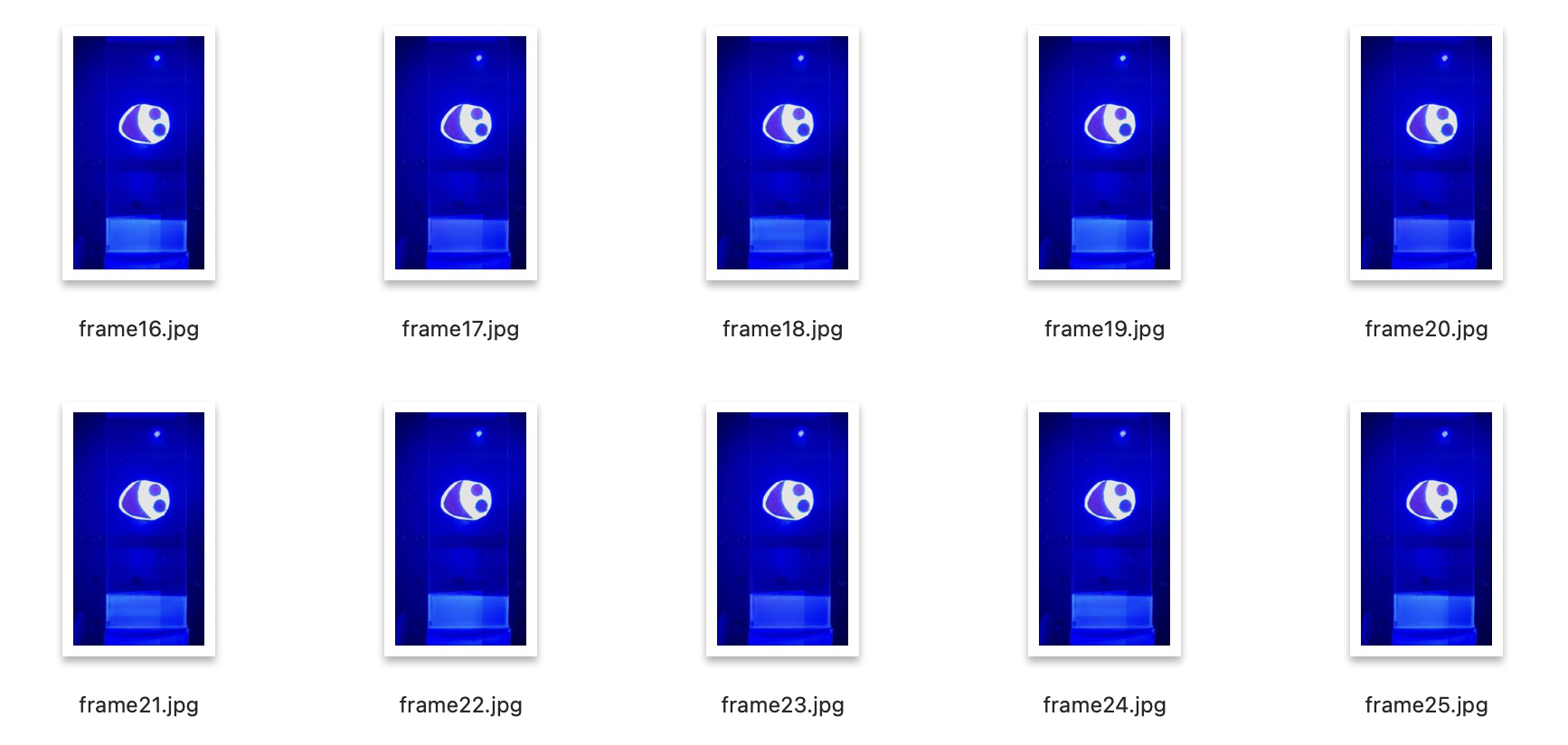


**Fig. S8. Frames from the video taken by iPhone 13 Pro (LED: 50 Hz, 40% duty; video frame rate: 30 fps).** To enable V-chopper detection, the shutter speed (S) needs to be faster than 0.012s (1/83.3 s), which is calculated by **Equation 3**. However, since there is no manual control of shutter speed on iPhone, the actual shutter speed may be slower. As a result, no gated frames have been captured with the V-chopper settings mentioned above.


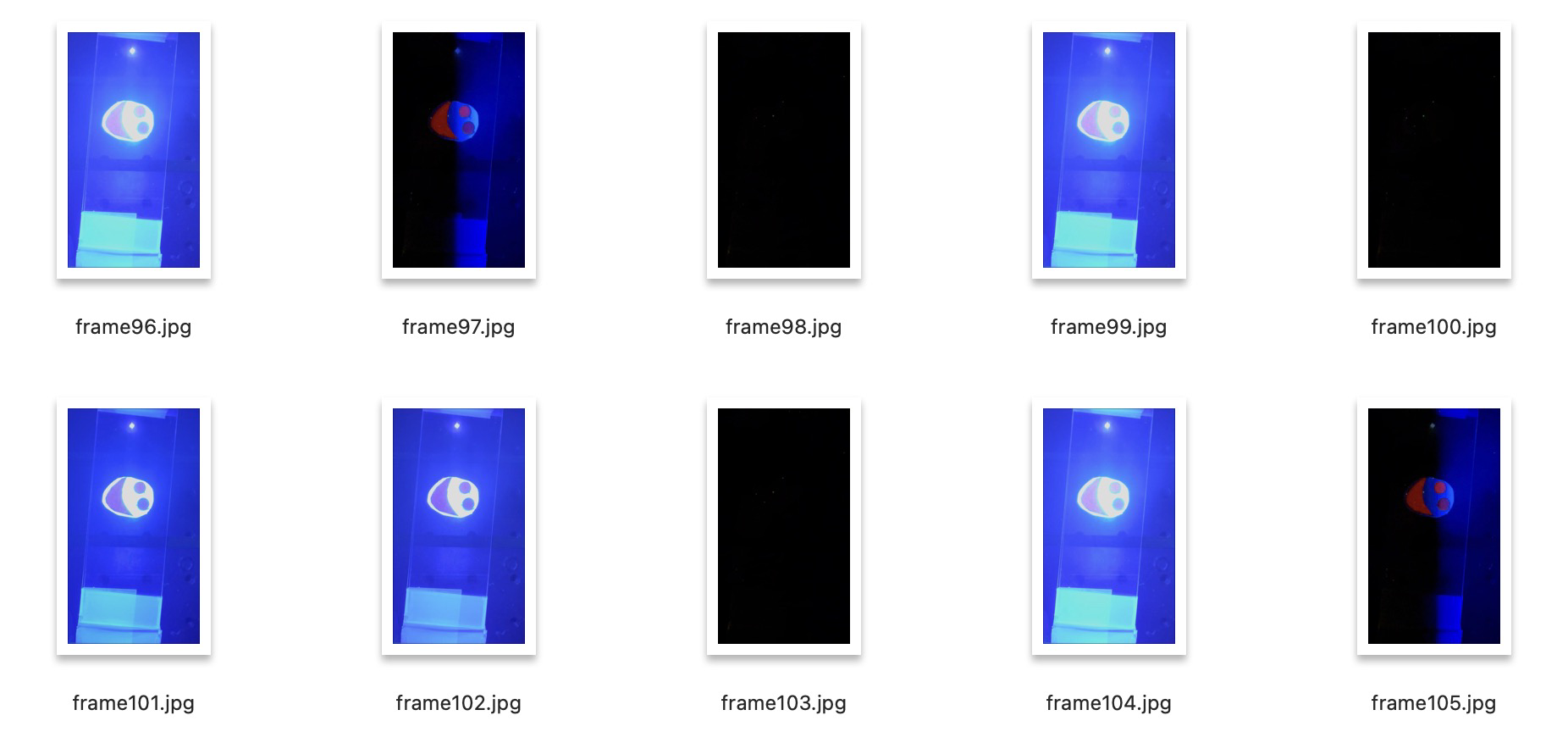


**Fig. S9. Frames from the video taken by iPhone 13 Pro (LED: 10 Hz, 20% duty; video frame rate: 30 fps).** LED settings are modified accordingly to meet **Equation 3**. However, the ERS effect is very obvious in the Frame #97 and #105.


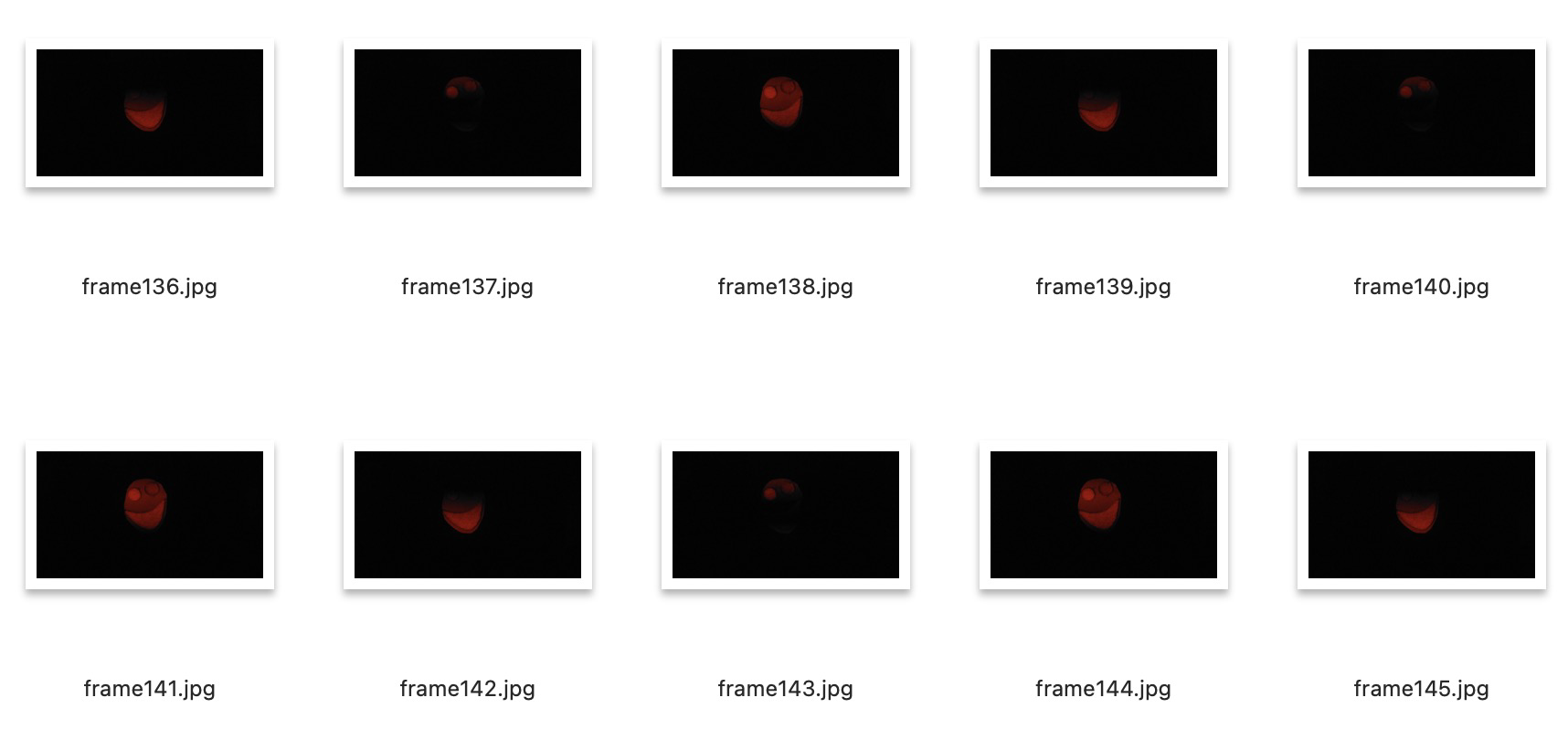


**Fig. S10. Frames from the video taken by LG V10 (LED: 50 Hz, 40% duty; video frame rate: 30 fps; shutter speed: 1/125 s).** The shutter speed can be manually controlled and was set according to **Equation 3**. However, the ERS effect is very severe and nearly 70% of the frames from the video are affected. This could be because LG V10 is a relatively old model and the ERS on this camera has a much longer readout time.


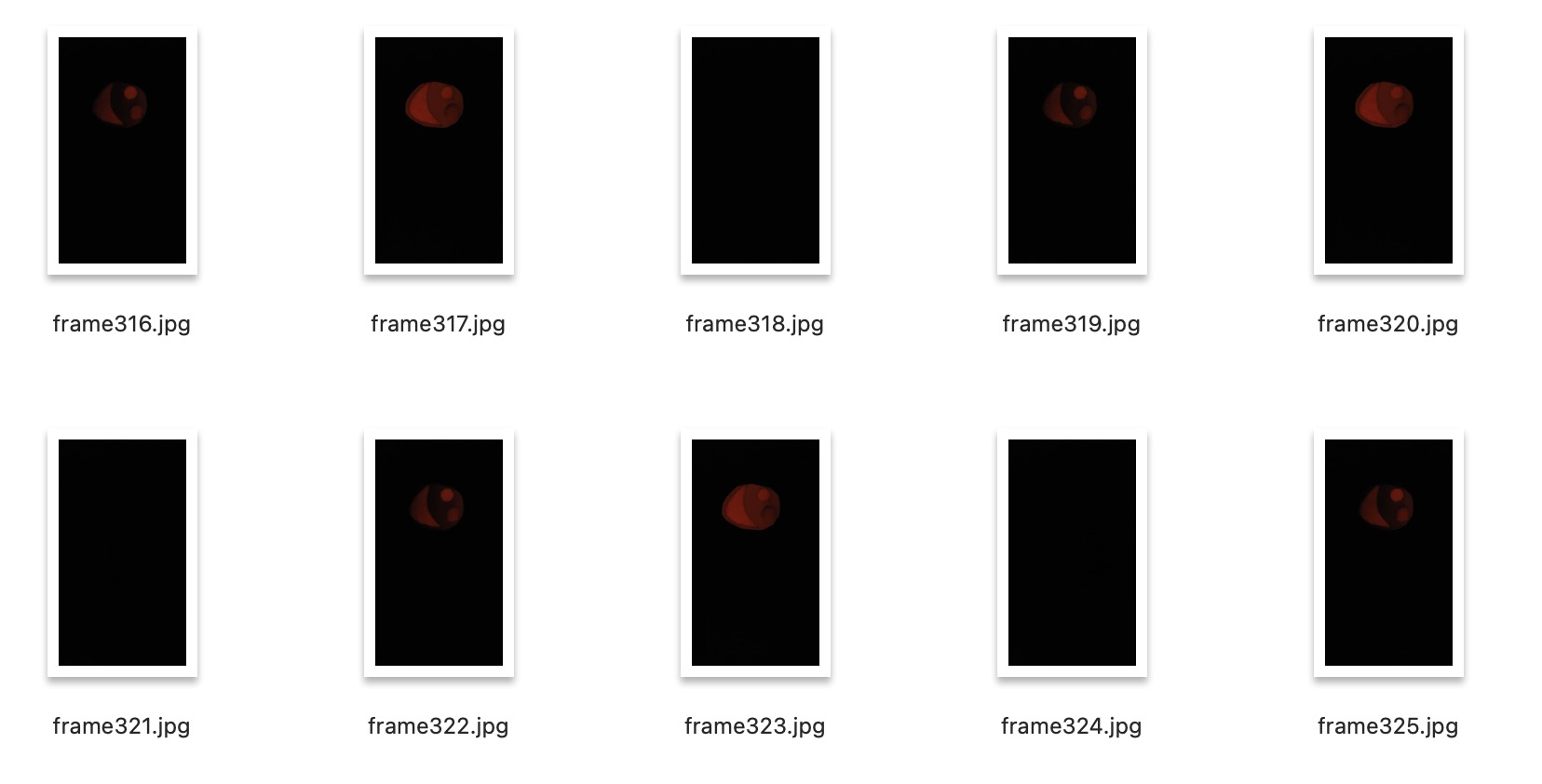


**Fig. S11. Frames from the video taken by Samsung Galaxy S9 (LED: 50 Hz, 40% duty; video frame rate: 30 fps; shutter speed: 1/350 s).** The shutter speed can be manually controlled and was set according to **Equation 3**. Frame #316, #319, #322 and #325 show very clear time-gated luminescent eyes and mouth without obvious autofluorescence from the face background. No obvious ERS effect was observed.

**Table S1. The CNN model complexity in terms of number of learnable parameters.**

| **Layer** | **Number of Learnable Parameters** |
| --- | --- |
| Convolution 1 | 160 |
| Batch Normalization 1 | 32 |
| Max Pooling 1 | 0 |
| Convolution 2 | 8224 |
| Batch Normalization 2 | 64 |
| Max Pooling 2 | 0 |
| Convolution 3 | 18496 |
| Batch Normalization 3 | 128 |
| Max Pooling 3 | 0 |
| Fully Connected Layer | 160100 |
| Batch Normalization 4 | 200 |
| Output Layer | 202 |
| ***Total Number of Learnable Parameters*** | ***187606*** |

**Table S2. List of Eu dyes for lifetime imaging used in this paper.**

| **Probe Dye** | **Labelled Lifetime** | **Calibrated Lifetime** | **Luminescence Color** | **Form** |
| --- | --- | --- | --- | --- |
| Calcium Sulfide | 1-4 Hours | 1.5 s | Red | Micron Particles |
| Strontium Aluminate Europium Dysprosium | 4-8 Hours | 5 s | Malachite Green | Micron Particles |
|  | 8-12 Hours | 7 s | Jade Green | Micron Particles |
|  | Over 12 Hours | 31 s | Cyan | Micron Particles |
| Tc-Eu | 100 µs | 76.6 ± 2.7 µs | Red | Powder |
| BHHCT-Eu | 250 µs | 167.2 ± 1.4 µs | Red | Powder |
| Eu Chelate Beads | 500 µs | 516.4 ± 7.0 µs | Red | Polystyrene Beads |

**Table S3. Recommended example settings of LED and V-chopper for different lifetime ranges on the smartphone.**

| **Lifetime** (*τ*) | **LED Frequency** (*F*)  **(40% duty)** | **V-Chopper** | |
| --- | --- | --- | --- |
|  |  | **Frame Rate** (*f*) | **Shutter Speed** (*s*) |
| 100 µs | 20 Hz | 30 fps | <1/500 s |
| 500 µs | 20 Hz | 30 fps | 1/350 s |
| 1 ms | 20 Hz | 30 fps | 1/350 s |
| 10 ms | 20 Hz | 30 fps | 1/350 s |
| 50 ms | 1 Hz | 60 fps | 1/350 s |
| 200 ms | 1 Hz | 60 fps | 1/350 s |
| 1s | ≤ 0.2 Hz | 60 fps | 1/350 s |

**Table S4. Comparison of our low-rate smartphone videoscopy with previously published works.**

| **Ref.** | **Probes** | **Lifetime Range** | **Gating Mechanism** | **Light Source** | **Delay Time** | **Smartphone Installation** | **Lifetime multiplexing** | **Sensing Type** |
| --- | --- | --- | --- | --- | --- | --- | --- | --- |
| 31 | Persistent luminescent crystals, CzDPS | Hundreds of milliseconds | Photograph after UV light off | External UV LED | NA | Standalone | No | Time-gated image |
| 32 | Persistent luminescent nanoparticles, SrAl2O4:Eu2+, Dy3+ | Longer than 1 sec | The first post-flash frame from the captured 30 fps video | Smartphone torch and flash | ~100 ms | Yes | No | Time-gated image |
| 33 | Phosphor with temperature-dependent lifetimes, Gd2O2S:Eu3+ | Hundreds of milliseconds | Fitting with gated frames from 30 fps videos | External UV LED, 375 nm | NA | No | No | Lifetime detection |
| 34 | Persistent luminescent crystals, CzDPS | Hundreds of milliseconds | Fitting with gated frames from 30 fps video | Integrated UV LED, 365 nm | NA | Yes | Yes | Lifetime image |
| 38 | Unconventional organic crystal, TBBU | Hundreds of milliseconds | Fitting with gated frames from 60 fps videos | External UV LED, 365 nm | 16.7 ms | Standalone | No | Lifetime image |
| 35 | Europium complexes,  Eu(TTA)3, TPEEu | 100 µs - 1 ms | Mechanical chopper | Integrated UV LED, 365 nm | 100 µs | Yes | Yes | Lifetime image |
| 36 | Upconversion nanocrystals,  Er/Yb doped NaYF4 and Tm/Yb doped NaYF4 | 100 µs - 1 ms | Map the luminescence in time domain to luminescence arc in spatial domain with a turntable | 980 nm CW laser | NA | Standalone | No | Lifetime detection |
| 37 | Europium complexes, Eu(TTA)3, Eu–tetracycline | 100 µs - 1 ms | Mechanical chopper | 405 nm laser | 33.3 µs, 66.7 µs | Standalone | No | Time-gated luminescence spectroscopy |
| 39 | PtTFPP/polystyrene film | 20-100 µs | Camera with electronic rolling shutter and lifetime resolved by frequency domain method | 405 nm laser | NA | Yes | Yes | Low-resolution lifetime image |
| *This work* | *Europium complexes (SrAl2O4:Eu2+, Dy3+, Tc-Eu, BHHCT-Eu and Eu chelate beads); Calcium sulfide* | *70 µs - seconds* | *V-chopping modulation with 30 or 60 fps videos* | *Integrated UV LED, 365 nm* | *11 µs minimum for 30 fps; 3 µs for 60 fps* | *Yes* | *Yes* | *Lifetime image* |

**Table S5. Lifetimes of letters in the “NCSU” pattern after UV excitation with different durations and irradiances.**

| **UV Duration** (*s*) | **UV Irradiance**  (*mW/cm^2^*) | **Lifetime** (*s*) | | | |
| --- | --- | --- | --- | --- | --- |
|  |  | **N** | **C** | **S** | **U** |
| 4 | 1 | 3.5 | 0.5 | 5.4 | 23.7 |
| 7 |  | 4.3 | 0.8 | 6.8 | 26.0 |
| 10 |  | 5.2 | 1.2 | 7.8 | 30.9 |
| 13 |  | 5.9 | 1.5 | 8.2 | 31.3 |
| 16 |  | 6.1 | 1.6 | 8.5 | 31.7 |
| 10 | 0.4 | 3.2 | 0.4 | 4.6 | 23.1 |
|  | 0.7 | 4.6 | 0.9 | 6.3 | 26.6 |
|  | 1 | 5.2 | 1.2 | 7.8 | 30.9 |
|  | 1.3 | 5.1 | 1.2 | 7.6 | 29.6 |
|  | 1.6 | 5.0 | 1.1 | 7.5 | 30.0 |

**Video V1 (separate file). Raw video recorded by Galaxy S9 smartphone for the detection of ultra-long lifetime in the seconds range.** The video was recorded at 60 fps with exposure time of 1/60 s. After the UV excitation, the letters of “N”, “C”, “S”, “U” emit glows for different time durations. The long luminescence decays are able to be captured and resolved by the 60 fps video frames directly in a single decay cycle. The video was played at a 4× speed to demonstrate the whole process of glowing from four letters.

**Video V2-3 (separate file). Raw videos (V2: 1× original speed, V3: 0.1× original speed) recorded by smartphone V-chopper device for lifetime measurement in the microsecond range.** The original video (V2) was recorded with a frame rate of 29.98 fps and exposure time of 1/350 s, meanwhile the LED was pulsed at 50 Hz with a 40% duty cycle. V2 was played at 1× speed, while V3 was played at a lower speed of 0.1× to clearly show the grated frames (zero background). These gated frames were then extracted by the CNN model as mentioned in the main text and used to resolve the lifetime image.

**Video V4 (separate file). Raw video (1× original speed) recorded by smartphone V-chopper device for multiplexed lifetime detection in the sub-hundred to hundreds of microseconds range.** The video (V4) was recorded with a frame rate of 29.98 fps and exposure time of 1/500 s, meanwhile the LED was pulsed at 30 Hz with a 40% duty cycle. The video started with LED-on frames and followed by sequential gated images from multiple decay cycles. As illustrated in **Figure 6a**, after the LED off, a series of gated frames (one gated image per cycle, m = 1) with increasing time delay $\Delta t_{n}$ were captured. The “Moon” (sub-hundred lifetime) disappeared very fast in two or three frames, but the “Wolf” with the longest lifetime is lasting much longer.
